# Supplementary material for: Sustainable Hues: Exploring the Molecular Palette of Biowaste Dyes through LC-MS Metabolomics
Source: Molecules. 2021 Nov 2;26(21):6645. doi: 10.3390/molecules26216645 (PMC8587104; doi:10.3390/molecules26216645)
Supplement: Supplementary file 1 [file molecules-26-06645-s001.zip › molecules-1440298-supplementary.pdf]

SUPPLEMENTARY MATERIAL

Sustainable Hues: Exploring the Molecular Palette of Biowaste Dyes through LC-MS Metabolomics

Ralph John Emerson J. Molino [1], Klidel Fae B. Rellin [1], Ricky B. Nellas [2] and Hiyas A. Junio [1,]*

[1] Secondary Metabolites Profiling Laboratory (SMPL), Institute of Chemistry, College of Science, University of the Philippines, Diliman, Quezon City 1101, Philippines ; rjmolino@up.edu.ph (R.J.E.J.M.); kbrellin@up.edu.ph (K.F.B.R.)

[2] Virtual Biochemical Simulations Laboratory (Good VIBEs), Institute of Chemistry, College of Science, University of the Philippines, Diliman, Quezon City 1101, Philippines; rbnellas@up.edu.ph

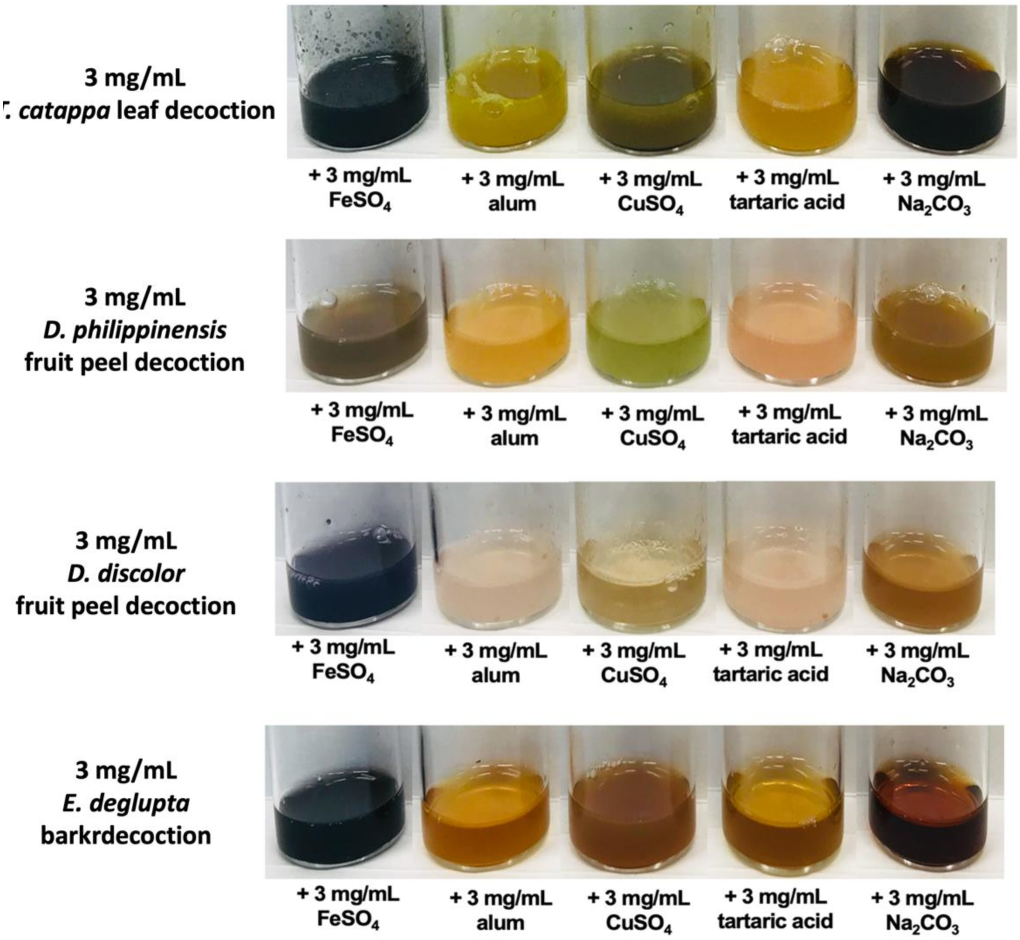

Figure S1. Dyebaths from the combination of biowaste colorants and mordant solutions. Image is a continuation of Figure 2b that contains the dyeing mixtures for *S. samarangense* and *S. malaccense.* Heated dyeing with cotton fabric yielded the color palette in Figure 3.

**Table S1. Colorimetric profile of the biowaste dyes measured in terms of Hue (H°), Saturation (%S), and Value (%V)**					
**Sample**	**+ 3 mg/mL $FeSO_4$**	**+3 mg/mL alum**	**+3 mg/mL $CuSO_4$**	**+ 3 mg/mL tartaric acid**	**+3 mg/mL Na_2CO_3**
Blank Solution (mordant only)	(30°, 52%, 97%)	(36°, 8%, 98%)	(84°, 5%, 95%)	(34°, 6%, 98%)	(40°, 8%, 99%)
+ 3mg/mL *T. catappa* leaf	(40°, 3%, 40%)	(43°, 50%, 85%)	(36°, 53%, 62%)	(32°, 34%, 85%)	(30°, 32%, 85%)
+ 3 mg/mL *D. philippinensis* fruit peel	(30°, 12%, 41%)	(40°, 50%, 76%)	(34°, 55%, 71%)	(48°, 63%, 86%)	(0°, 21%, 78%)
+ 3 mg/mL *E. deglupta* bark	(20°, 6%, 21%)	(28°, 21%, 80%)	(20°, 56%, 53%)	(18°, 44%, 76%)	(16°, 48%, 58%)
+ 3 mg/mL *D. blancoi* fruit peel	(28°, 18%, 54%)	(24°, 29%, 72%)	(20°, 41%, 73%)	(24°, 20%, 84%)	(18°, 26%, 70%)
+ 3 mg/mL *S. malaccense* fruit peel	(24°, 18%, 63%)	(20°, 14%, 78%)	(24°, 27%, 70%)	(46°, 42%, 83%)	(26°, 17%, 92%)
+ 3 mg/mL *S. samarangense* fruit peel	(30°, 6%, 49%)	(26°, 18%, 84%)	(30°, 39%, 76%)	(28°, 17%, 87%)	(26°, 23%, 93%)

Table S2. List of putatively identified metabolites from aqueous dye from *T. catappa* leaf							
	Compound Name	t_R (mins.)	Major Ion	Accurate Mass	Exact Mass	ppm error	Cosine
1	Sucrose	0.75	$[M+Na]^+$	365.1051	365.1060	2.41	0.82
2	Adenosine	0.8	$[M+H]^+$	268.1046	268.1040	2.13	0.91
3	Isoorientin	4.03	$[M+H]^+$	449.1065	449.1073	1.76	0.94
4	Isovitexin	4.19	$[M+H]^+$	433.1129	433.1129	0.05	0.95
5	Kaempferol-3-O-glucoside	4.57	$[M+H]^+$	449.1065	449.1073	1.76	0.9
6	Quercetin	5.1	$[M+H]^+$	303.0497	303.0499	0.76	0.87
7	Dilinolenin	8.13	$[M+H]^+$	613.4847	613.4827	3.34	0.77
8	Pheophytin A	9.11	$[M+H]^+$	871.5757	871.5732	2.87	0.83

**Table S3. List of putatively identified metabolites from aqueous dye from *D. philippinensis* fruit peel**							
	**Compound Name**	**t_R (mins.)**	**Major Ion**	**Accurate Mass**	**Exact Mass**	**ppm error**	**Cosine**
1	Citrulline	0.68	$[M+H]^+$	176.1022	176.1030	4.37	0.96
2	(S)-Dihydroorotate	0.68	$[M+H]^+$	159.0409	159.0403	3.77	0.8
3	Sucrose	0.75	$[M+Na]^+$	365.1051	365.1060	2.41	0.82
4	Adenosine	0.96	[M+H]+	268.1046	268.1040	2.13	0.96
5	5-Methylthioadenosine	1.27	[M+H]+	298.0962	298.0968	2.15	0.94
6	Tryptophan	1.39	$[M+H-NH_3]$+	188.0699	188.0706	3.72	0.83
7	Procyanidin B2	3.05	$[M+H]^+$	579.1483	579.1497	2.42	0.8
8	Epicatechin	3.35	$[M+H]^+$	291.0869	291.0863	2.03	0.92
9	Catechin	3.6	$[M+H]^+$	291.0869	291.0863	2.03	0.85
10	Vitexin	4.1	$[M+H]^+$	433.1129	433.1129	0.05	0.92
11	Vitexin	4.11	$[M+H]^+$	433.1129	433.1129	0.05	0.89
12	Isovitexin	4.2	$[M+H]^+$	433.1129	433.1129	0.05	0.9
13	Epicatechin Gallate	4.25	$[M+H]^+$	443.0954	443.0973	4.22	0.92
14	Quercetin-3-O-glucuronide	4.81	$[M+H]^+$	479.0843	479.0820	4.76	0.9
15	Tiliroside	5	$[M+H]^+$	595.1417	595.1446	4.91	0.95
16	Quercetin	5.1	$[M+H]^+$	303.0497	303.0499	0.76	0.91
17	Kaempferol	5.39	$[M+H]^+$	287.0551	287.0550	0.31	0.89
18	PC(18:2/0:0)	6.25	$[M+H]^+$	520.3402	520.3398	0.83	0.89

19	PC(16:0)	6.44	$[M+H]^+$	496.3399	496.3398	0.26	0.89
20	PC(16:0/18:2)	7.52	$[M+H]^+$	758.5696	758.5694	0.22	0.96
21	PC(16:0/18:1)	8	$[M+H]^+$	760.5828	760.5851	3.00	0.97
22	Dilinolenin	8.13	$[M+H]^+$	613.4847	613.4827	3.34	0.74
23	PC(18:0/18:2)	8.21	$[M+H]^+$	786.6000	786.6007	0.93	0.94
24	PE(16:0/18:1)	8.32	$[M+Na]^+$	740.5215	740.5206	1.19	0.82
25	PE(16:0/18:2)	8.34	$[M+H]^+$	716.5249	716.5225	3.38	0.82
26	PC(18:3/18:3)	8.97	$[M+H]^+$	778.5411	778.5381	3.81	0.91
27	PC(18:2/18:2)	9.05	$[M+H]^+$	782.5711	782.5694	2.13	0.96

Table S4. List of putatively identified metabolites from aqueous dye from *S. malacensse* fruit peel.

	Compound Name	t_R (mins.)	Major Ion	Accurate Mass	Exact Mass	ppm error	Cosine
1	Cyanidin-3-O-Glucoside	1.05	$[M]^+$	449.1065	449.1078	2.98	0.97
2	Kaempferol-3-O-Glucoside	1.05	$[M+H]^+$	449.1065	449.1078	2.98	0.97
3	Cyanidin-3,5-O-Diglucoside	1.05	$[M]^+$	611.1608	611.1607	0.23	0.87
4	Cyanidin	1.05	$[M]^+$	287.0551	287.0550	0.31	0.77
5	Quercetin	5.1	$[M+H]^+$	303.0497	303.0499	0.76	0.83
6	Oleanolic acid	7.83	$[M+H-H2O]^+$	439.3563	439.3571	1.73	0.72
7	9-Octadecenamide	7.92	$[M+H]^+$	282.2784	282.2791	2.62	0.81

Table S5. List of putatively identified metabolites from aqueous dye from *S. samarangense* fruit peel.

	Compound Name	t_R (mins.)	Major Ion	Accurate Mass	Exact Mass	ppm error	Cosine
1	Adenosine	0.8	$[M+H]^+$	268.1046	268.1040	2.13	0.96
2	Cyandin-3-O-glucoside	1.05	$[M]^+$	449.1065	449.1078	2.98	0.94
3	5-Methylthioadenosine	1.27	$[M+H]^+$	298.0962	298.0968	2.15	0.94
4	Catechin	3.73	$[M+H]^+$	291.0869	291.0863	2.03	0.85
5	Myricetin	4.48	$[M+H]^+$	319.0433	319.0448	4.83	0.87
6	Isoquercetin	4.54	$[M+H]^+$	465.1033	465.1028	1.18	0.96
7	Kaempferol-3-O-glucoside	4.57	$[M+H]^+$	449.1065	449.1073	1.76	0.95
8	Quercetin-3O-arabinofuranoside (Avicularin)	4.7	$[M+H]^+$	435.0909	435.0922	2.96	0.94
9	Isorhamnetin 3-O-glucoside	4.7	$[M+H]^+$	479.1201	479.1184	3.55	0.90
10	Isorhamnetin	4.94	$[M+H]^+$	317.0656	317.0656	0.06	0.84

11	Quercetin	5.1	[M+H]$^+$	303.0497	303.0499	0.76	0.91
12	Alpinetin	5.56	[M+H]$^+$	271.0955	271.0965	3.65	0.92
13	Alpinetin Methyl Ether	5.78	[M+H]$^+$	285.1122	285.1121	0.21	0.92
14	PC(18:2/0:0)	6.25	[M+H]$^+$	520.3402	520.3398	0.83	0.89
15	PC(16:0/0:0)	6.44	[M+H]$^+$	496.3399	496.3398	0.26	0.85
16	Angoletin	7.42	[M+H]$^+$	301.142	301.1434	4.78	0.86
17	PC(16:0/18:2)	7.52	[M+H]$^+$	758.5696	758.5694	0.22	0.96
18	4',6'-Dimethoxy-2'-hydroxychalcone	7.55	[M+H]$^+$	285.1122	285.1121	0.21	0.92
19	2,4,6-Trimethoxychalcone	7.61	[M+H]$^+$	299.1286	299.1278	2.71	0.90
20	Oleanolic acid	7.83	[M+H-H2O]$^+$	439.3563	439.3571	1.73	0.72
21	9-Octadecenamide	7.92	[M+H]$^+$	282.2784	282.2791	2.62	0.8
22	PC(16:0/18:1)	8.00	[M+H]$^+$	760.5828	760.5851	3.00	0.97
23	PE(16:0/18:2)	8.34	[M+H]$^+$	716.5249	716.5225	3.38	0.82
24	PE(18:2/18:2)	8.58	[M+H]$^+$	740.5215	740.5225	1.32	0.78

Table S6. List of putatively identified metabolites from aqueous dye from *E. deglupta* bark

	**Compound Name**	**t_R (mins.)**	**Major Ion**	**Accurate Mass**	**Exact Mass**	**ppm error**	**Cosine**
1	Sucrose	0.68	[M+Na]$^+$	365.1051	365.10598	2.41	0.82
2	Melezitose	0.68	[M+Na]$^+$	527.1578	527.1588	1.90	0.89
3	Procyanidin B2	3.06	[M+H]$^+$	579.1483	579.1497	2.42	0.8
4	Epicatechin	3.3	[M+H]$^+$	291.0869	291.08631	2.03	0.95
5	Catechin	3.5	[M+H]$^+$	291.0869	291.08631	2.03	0.95
6	13-Docosenamide	7.55	[M+H]$^+$	338.34174	338.3416	0.41	0.87

Table S7. List of putatively identified metabolites from aqueous dye from *D. blancoi* fruit peel

	**Compound Name**	**t_R (mins.)**	**Major Ion**	**Accurate Mass**	**Exact Mass**	**ppm error**	**Cosine**
1	Melezitose	0.75	[M+Na]$^+$	527.1578	527.1588	1.90	0.89
2	Sucrose	0.75	[M+Na]$^+$	365.1051	365.10598	2.41	0.82
3	Cyandin-3-O-diglucoside	0.82	[M]$^+$	449.1065	449.10784	2.98	0.97
4	Kaempferol-3-O-glucoside	3.74	[M]$^+$	449.1065	449.10784	2.98	0.97

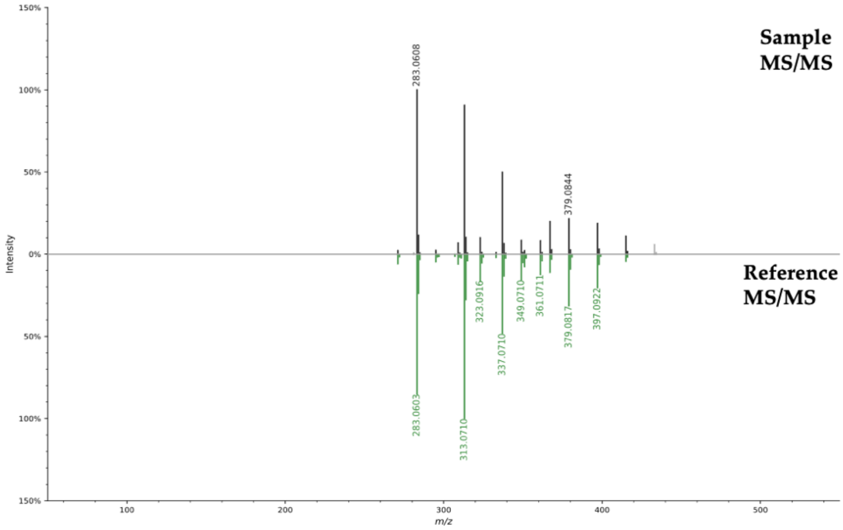

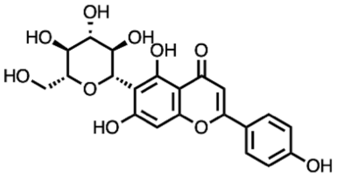

Spectral Match to Isovitexin
Cosine: 0.97, ppm error: 0.05

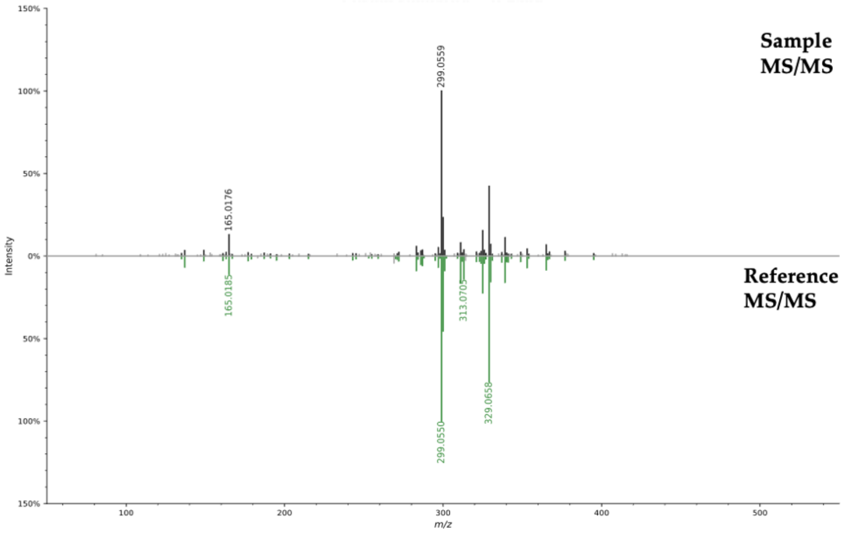

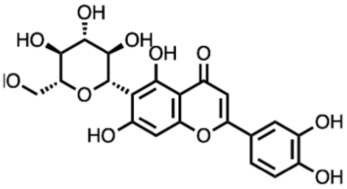

Spectral Match to Isoorientin
Cosine: 0.94, ppm error: 1.75

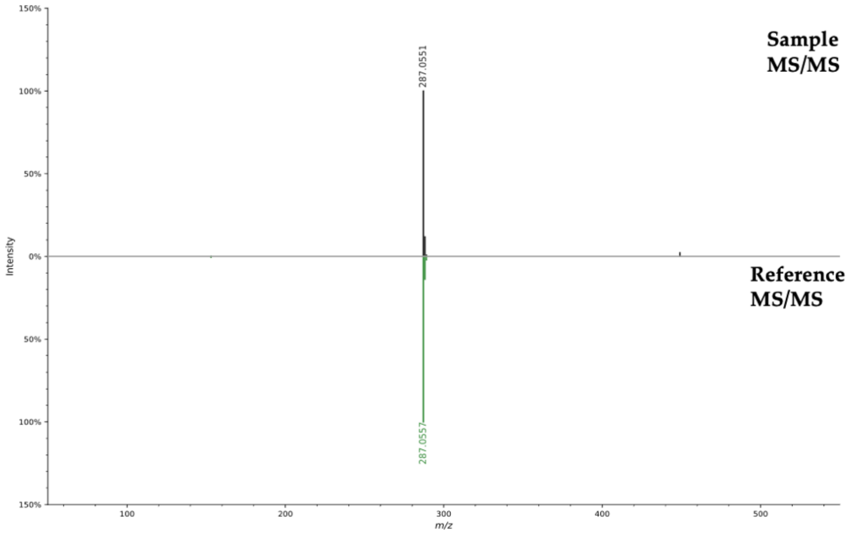

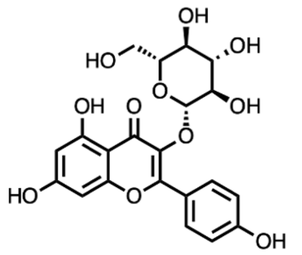

Spectral Match to Kaempferol-3O-glucoside
Cosine: 0.90, ppm error: 1.75

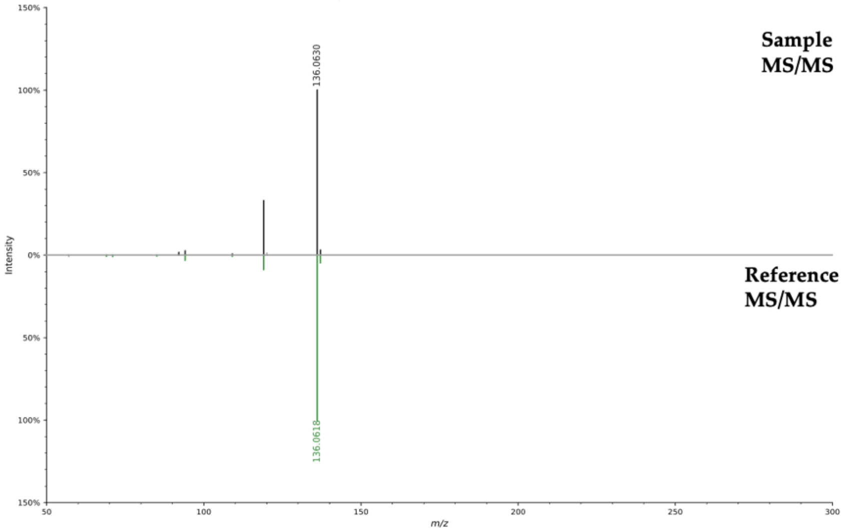

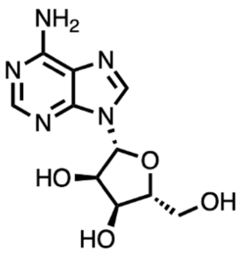

Spectral Match to Adenosine
Cosine: 0.91, ppm error: 0.80

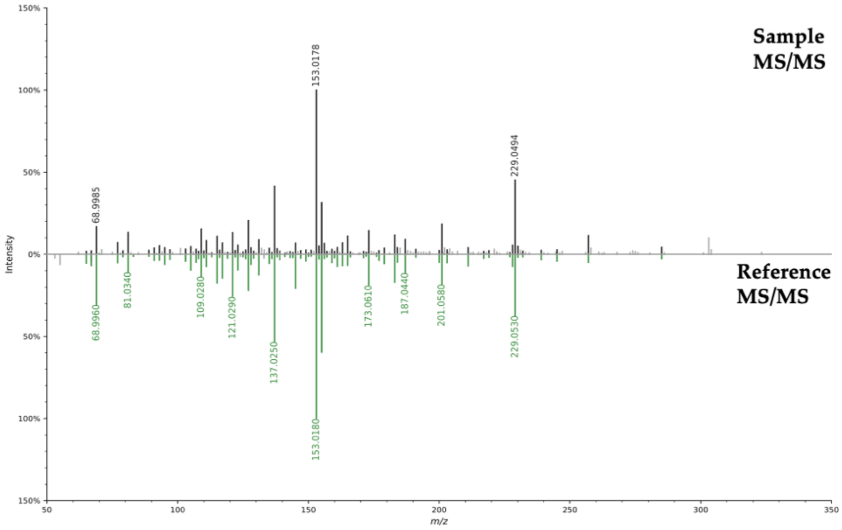

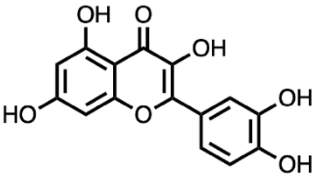

Spectral Match to Quercetin
Cosine: 0.87, ppm error: 0.76

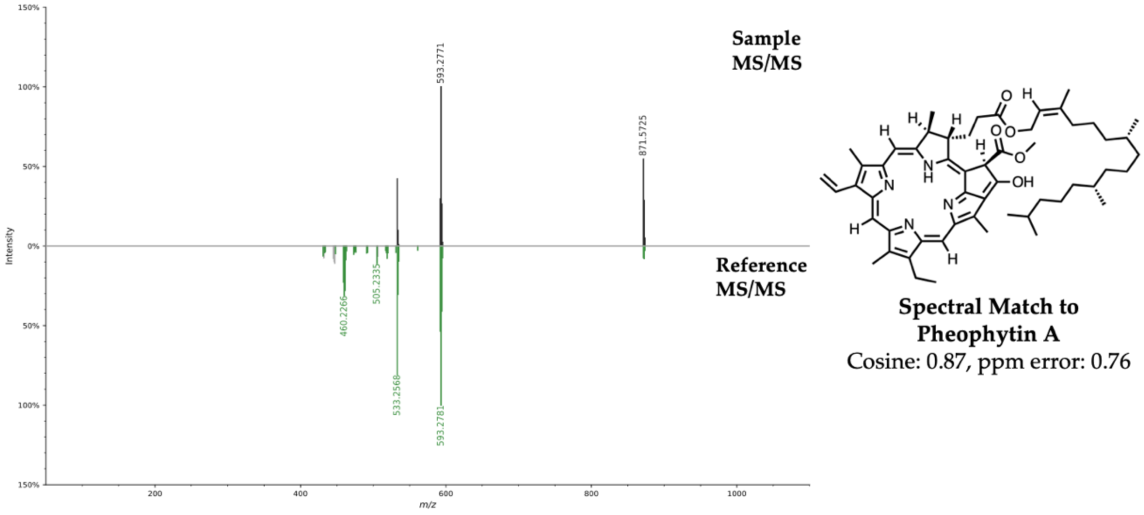

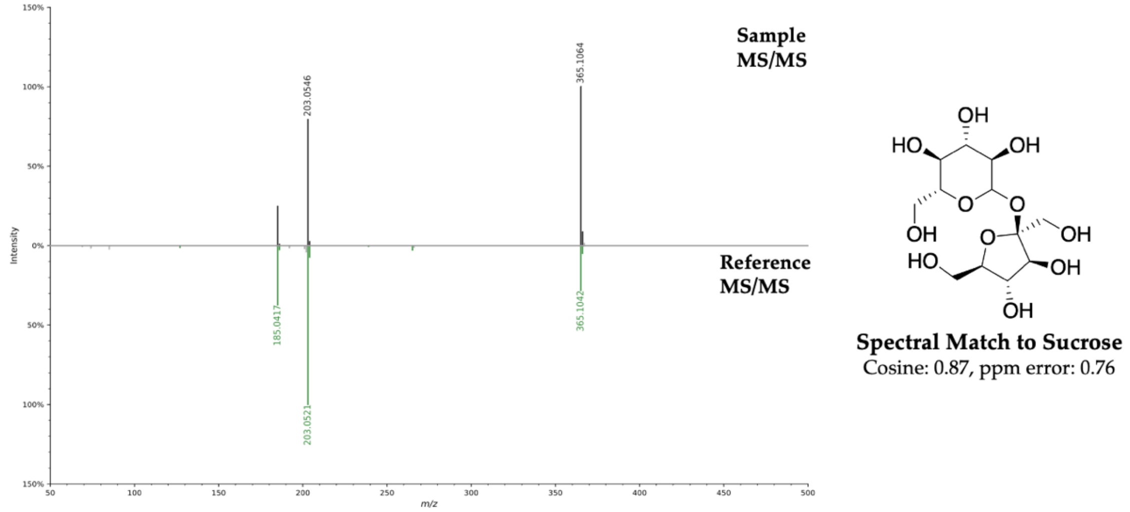

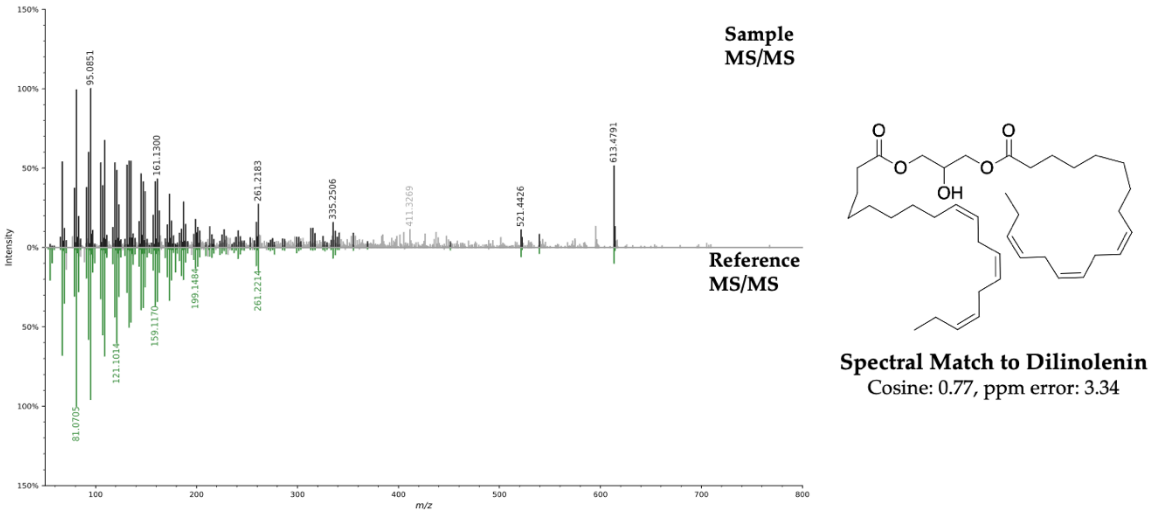

Figure S2. Tail-to-tail alignment of putatively identified metabolites from aqueous extract of *T. catappa* leaf.

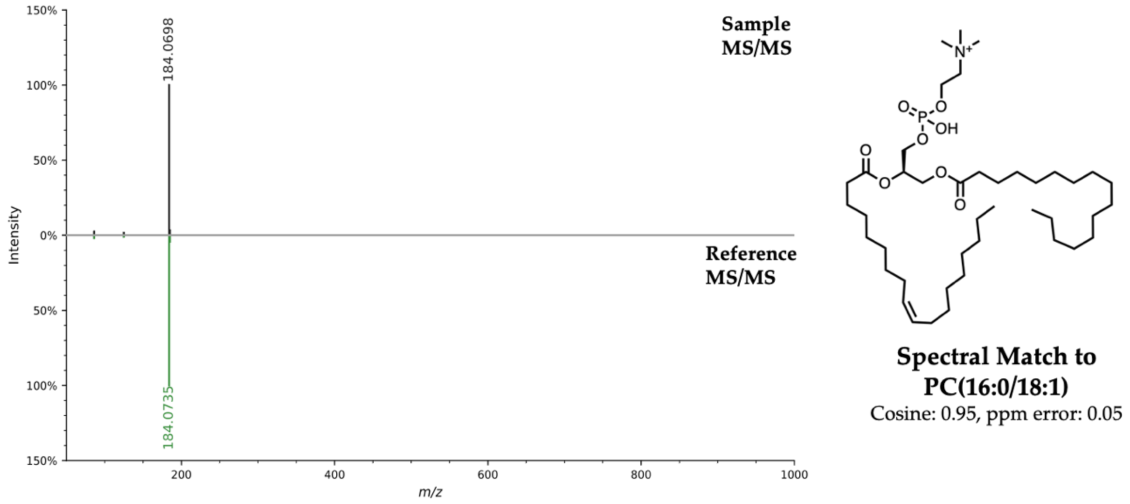

Sample MS/MS
Reference MS/MS
184.0698
184.0735
Intensity
m/z
Spectral Match to PC(16:0/18:1)
Cosine: 0.95, ppm error: 0.05

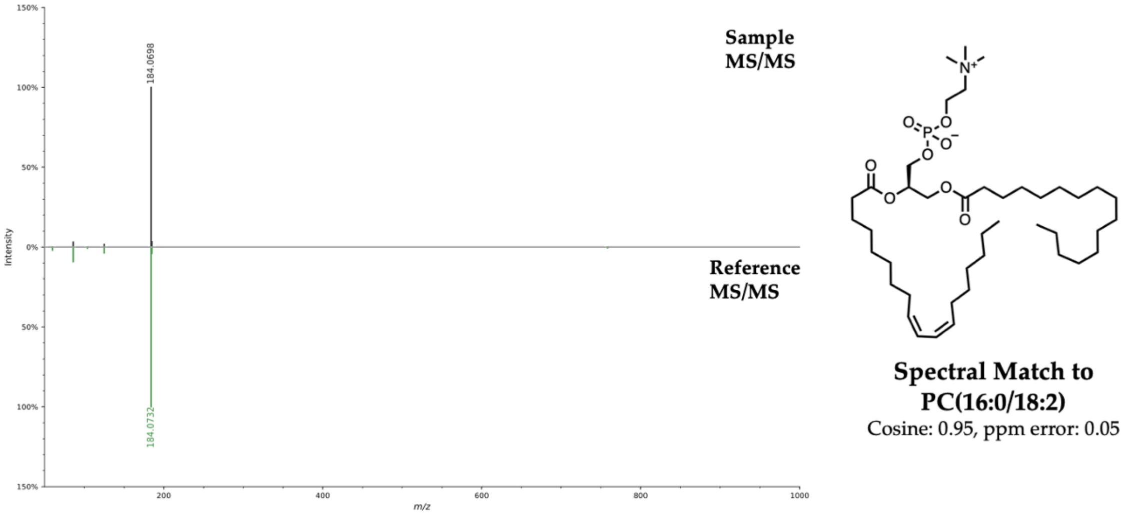

Sample MS/MS
Reference MS/MS
184.0698
184.0732
Intensity
m/z
Spectral Match to PC(16:0/18:2)
Cosine: 0.95, ppm error: 0.05

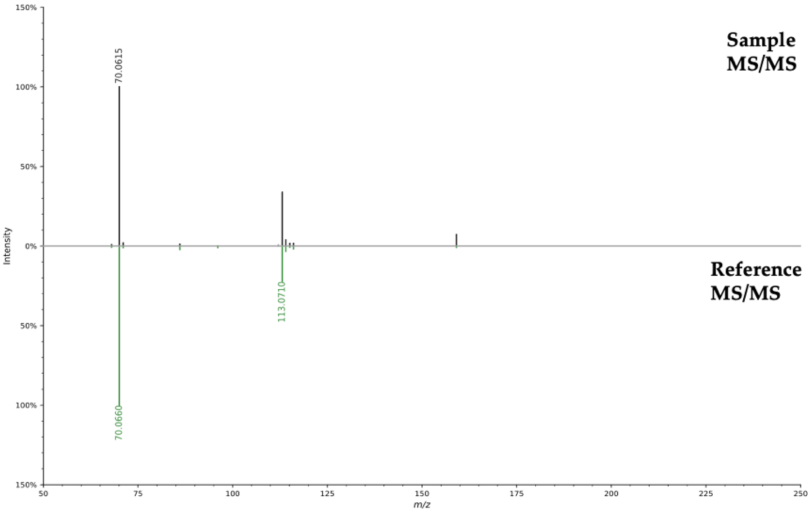

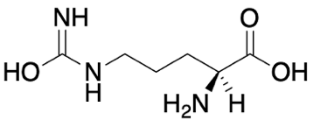

Spectral Match to Citrulline
Cosine: 0.96, ppm error: 4.36

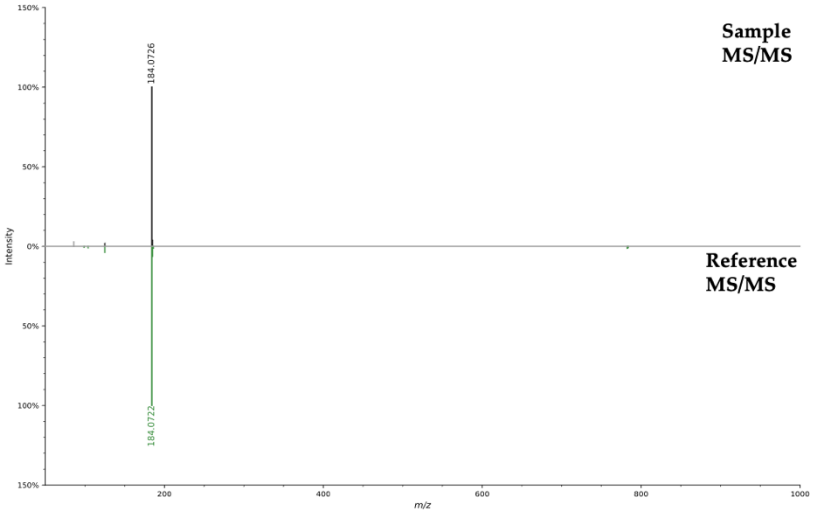

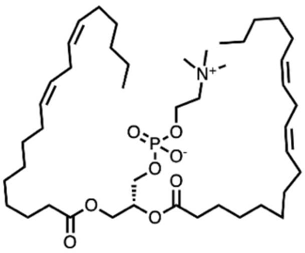

Spectral Match to PC(18:2/18:2)
Cosine: 0.96, ppm error: 4.36

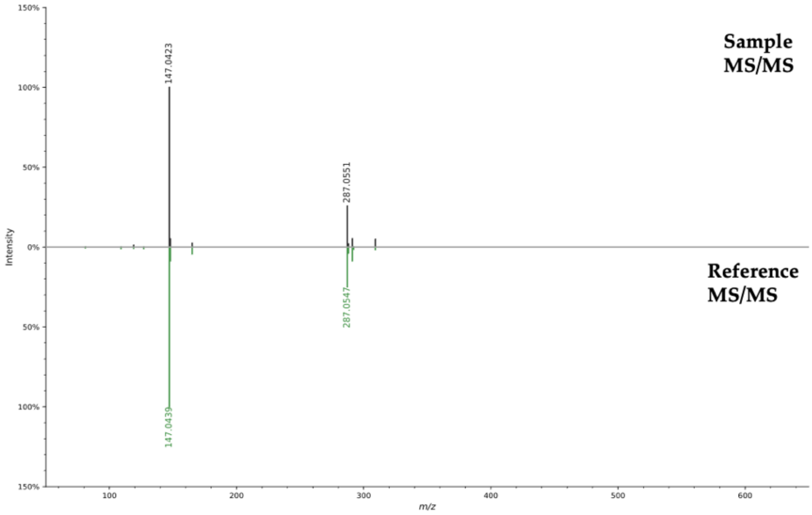

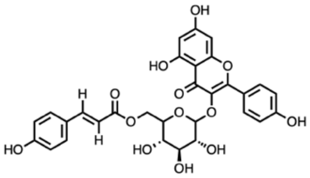

Spectral Match to Tiliroside
Cosine: 0.96, ppm error: 4.36

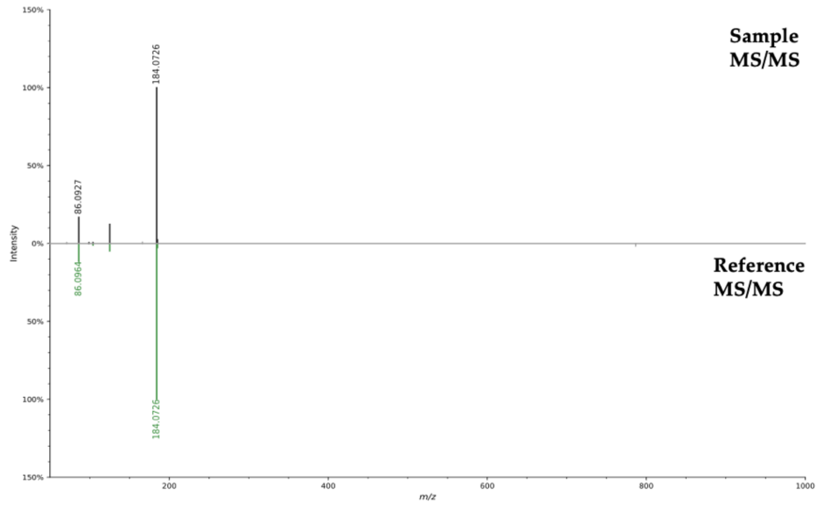

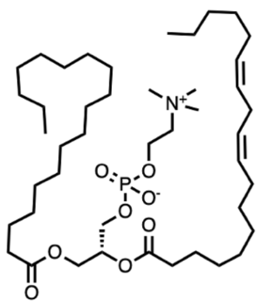

Spectral Match to PC(18:0/18:2)
Cosine: 0.94, ppm error: 0.92

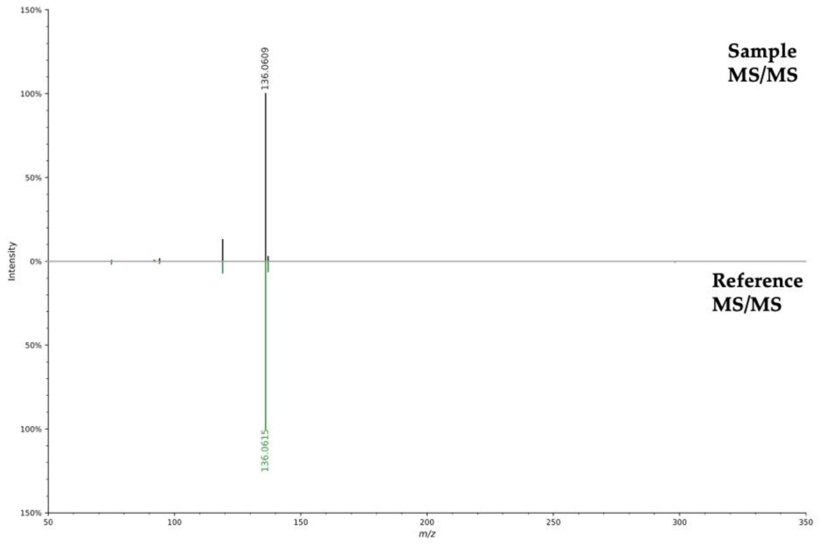

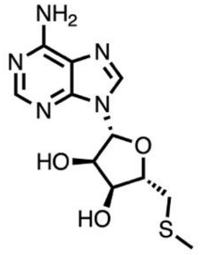

Spectral Match to 5-Deoxy-5'-methylthioadenosine
Cosine: 0.94, ppm error: 2.15

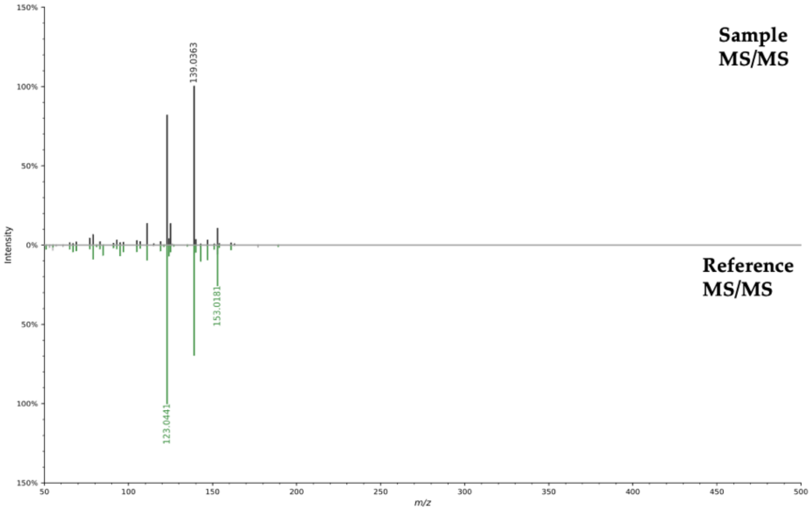

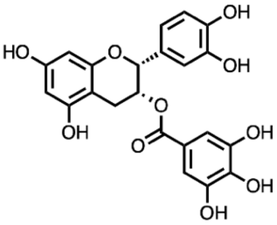

Spectral Match to Epicatechin Gallate
Cosine: 0.92, ppm error: 4.22

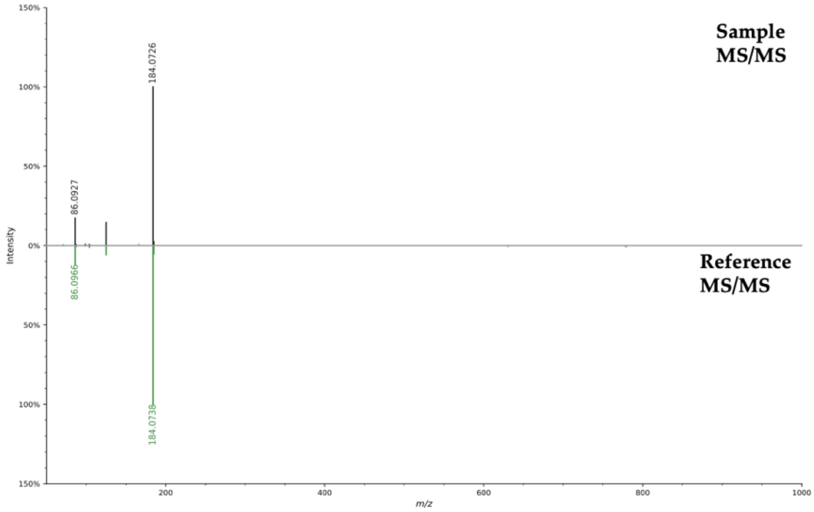

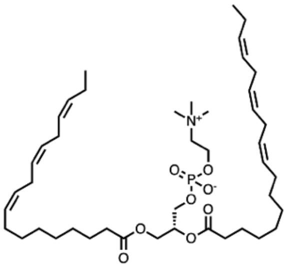

Spectral Match to PC(18:3/18:3)
Cosine: 0.92, ppm error: 4.22

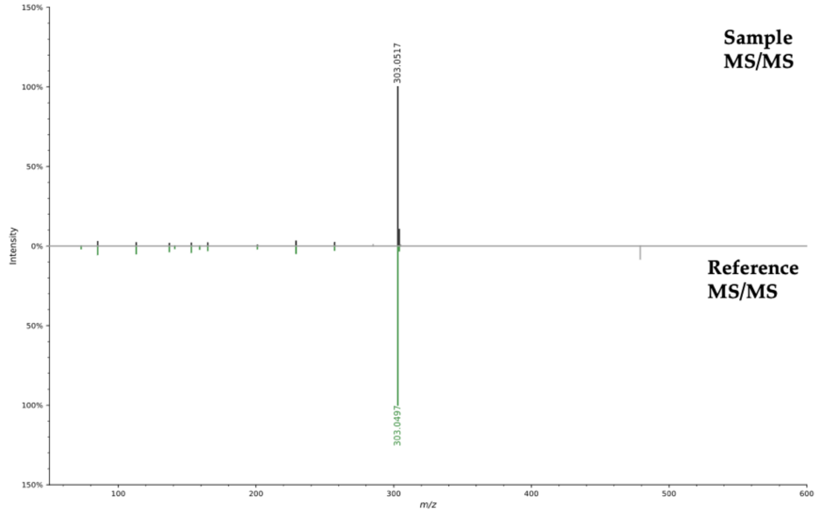

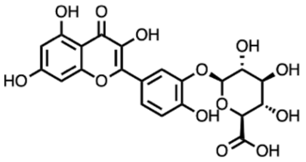

Spectral Match to
Quercetin-O-glucuronide
Cosine: 0.90, ppm error: 4.76

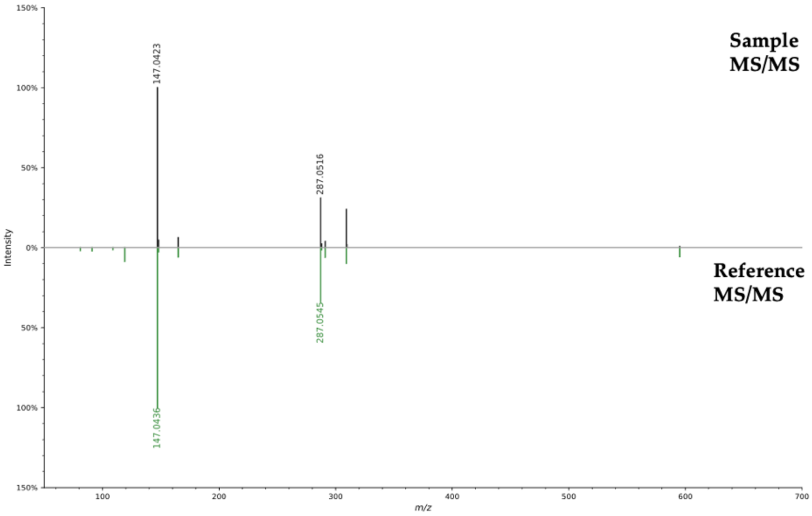

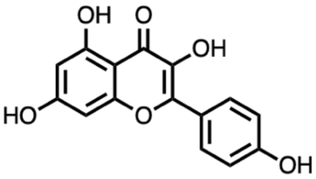

Spectral Match to
Kaempferol
Cosine: 0.89, ppm error: 0.31

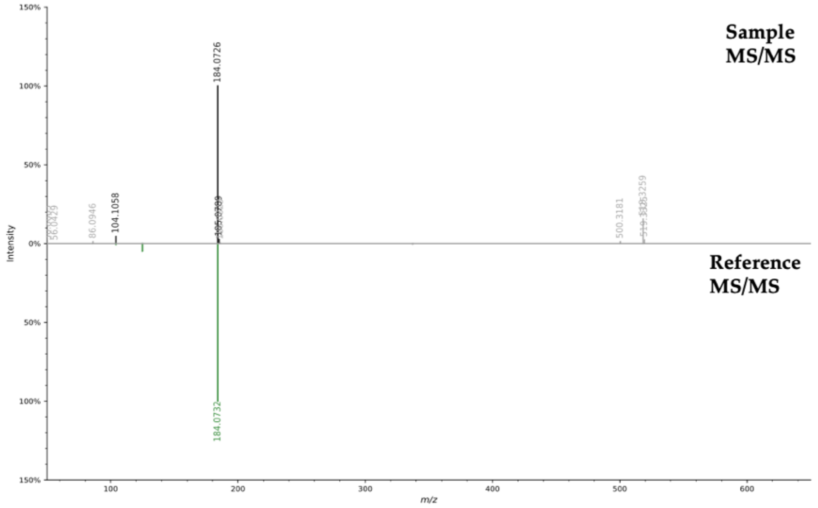

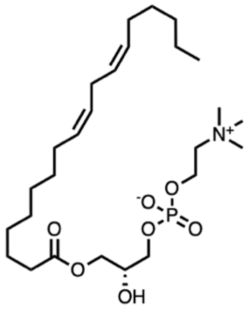

Spectral Match to
PC(18:2/0:0)
Cosine: 0.89, ppm error: 0.31

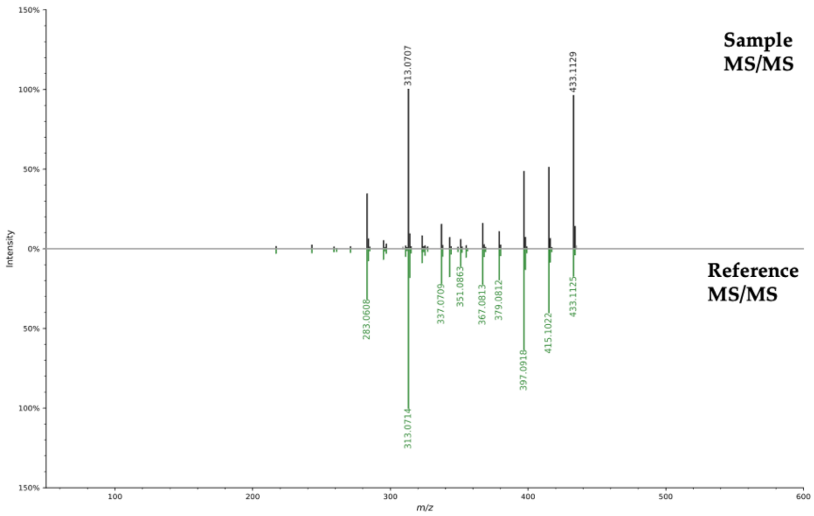

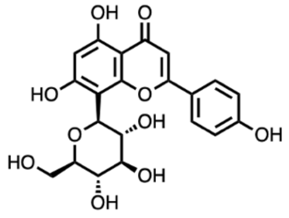

Spectral Match to Vitexin
Cosine: 0.89, ppm error: 0.05

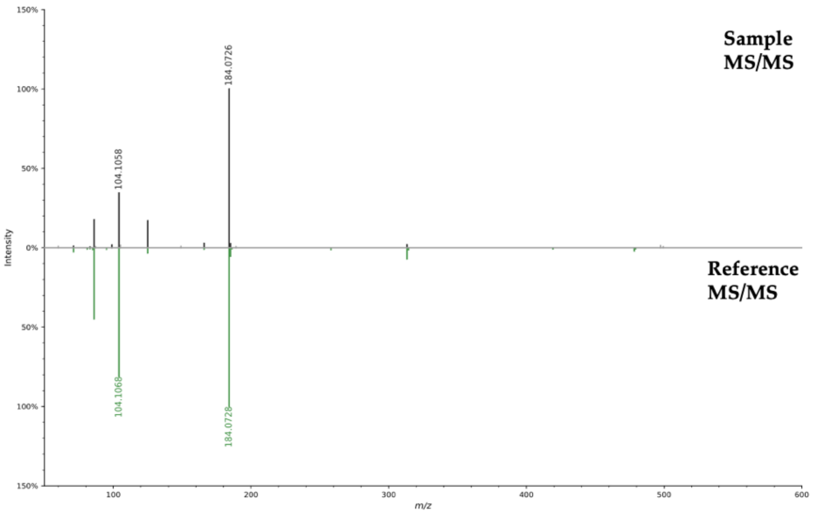

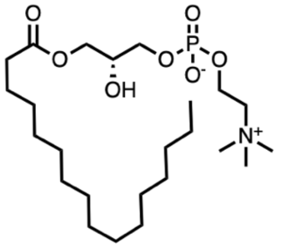

Spectral Match to PC(16:0)
Cosine: 0.89, ppm error: 0.05

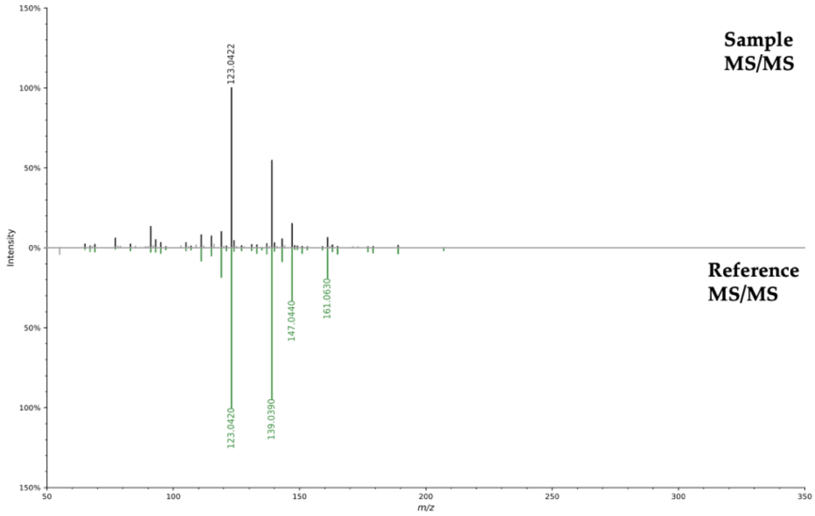

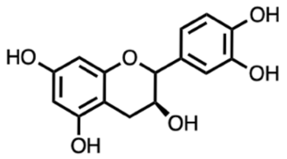

Spectral Match to Catechin
Cosine: 0.85, ppm error: 2.02

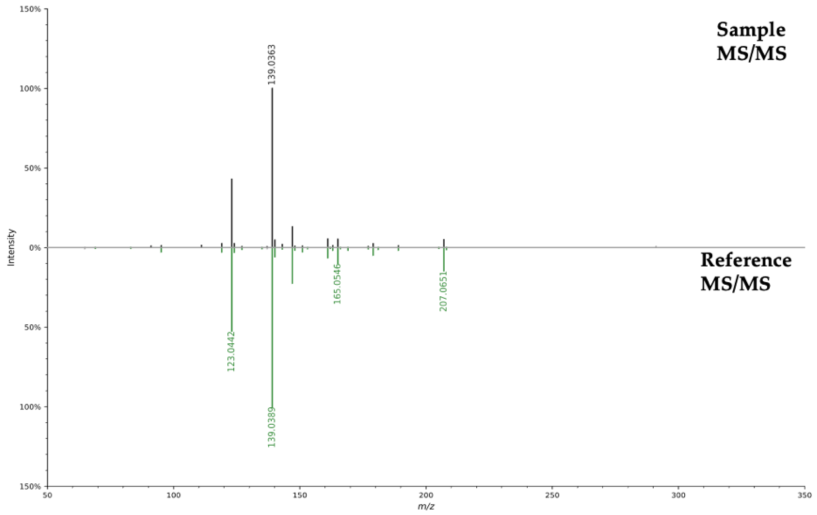

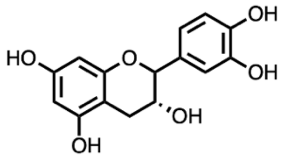

Spectral Match to Epicatechin
Cosine: 0.92, ppm error: 2.02

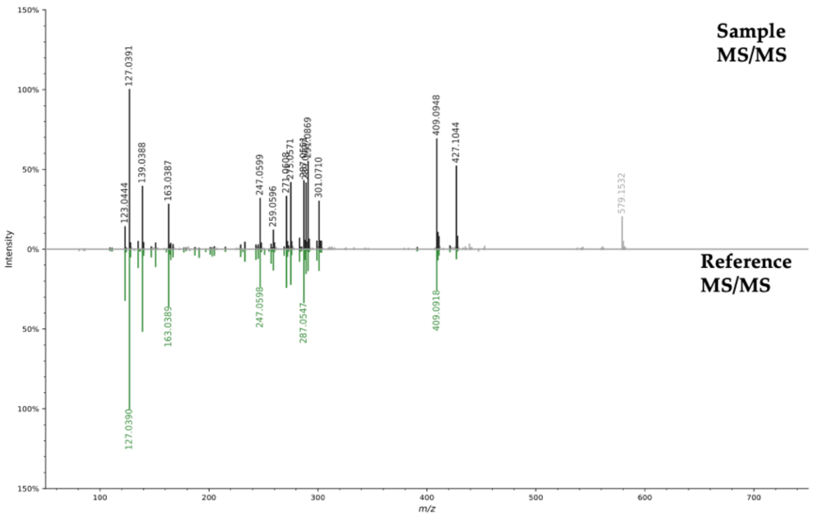

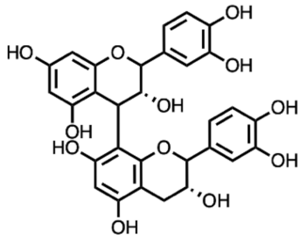

Spectral Match to Procyanidin B2
Cosine: 0.80, ppm error: 2.42

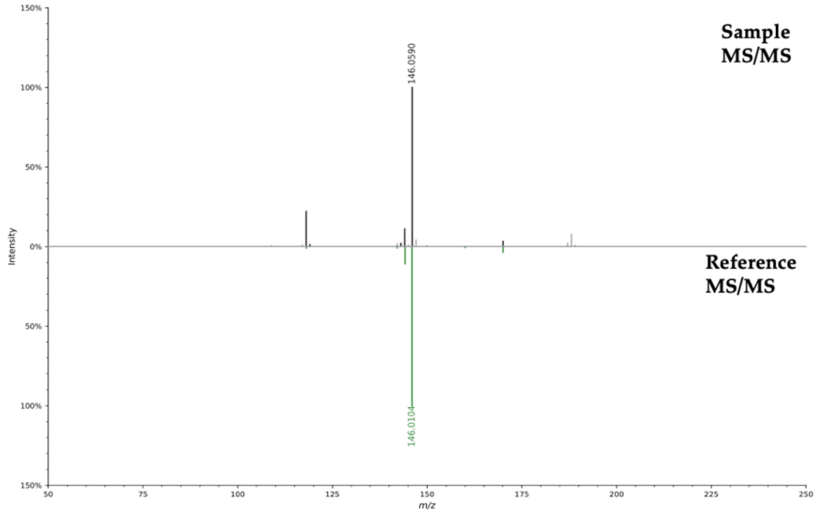

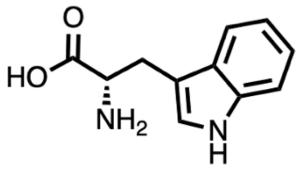

Spectral Match to Tryptophan
Cosine: 0.80, ppm error: 2.42

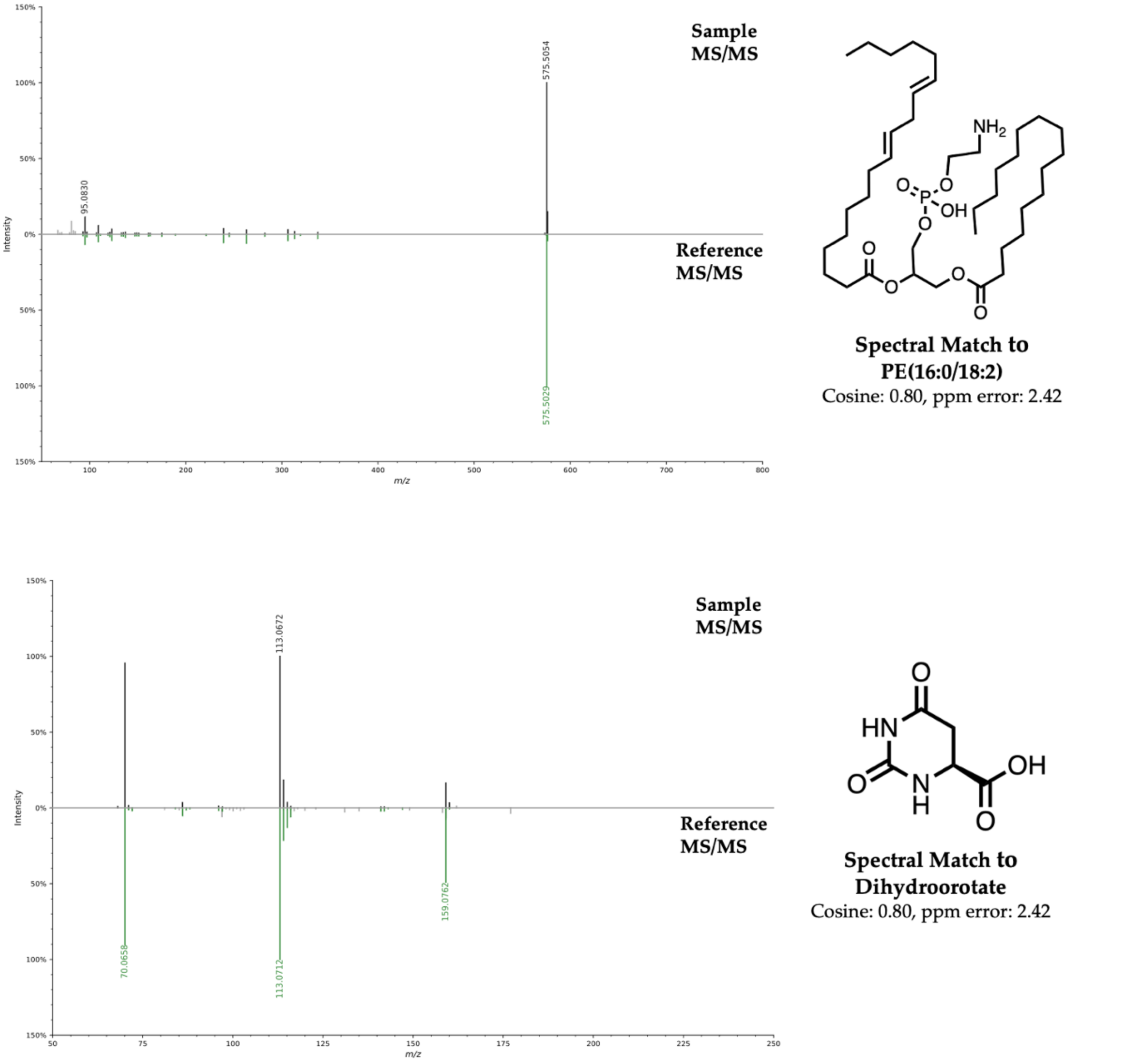

Figure S3. Tail-to-tail alignment of putatively identified metabolites from aqueous extract of *D. philippinensis* fruit peel.

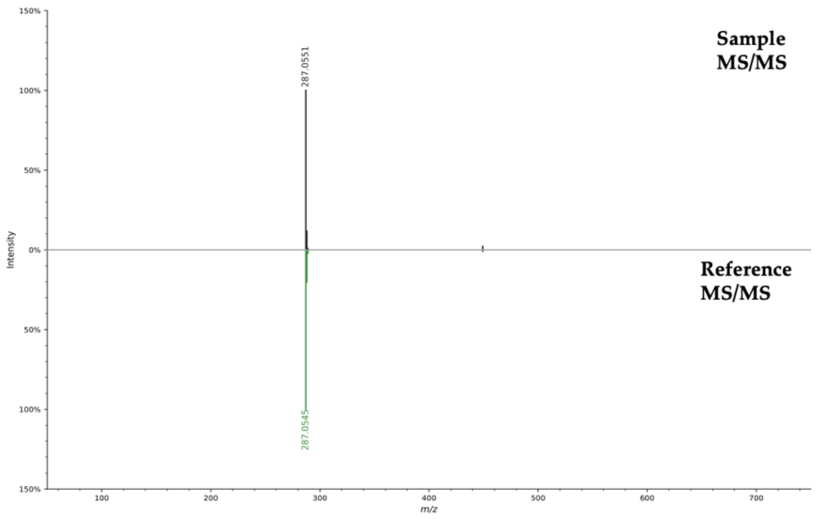

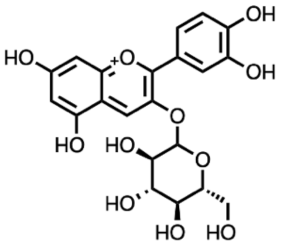

Spectral Match to Cyanidin-3-O-Glucoside
Cosine: 0.97, ppm error: 2.98

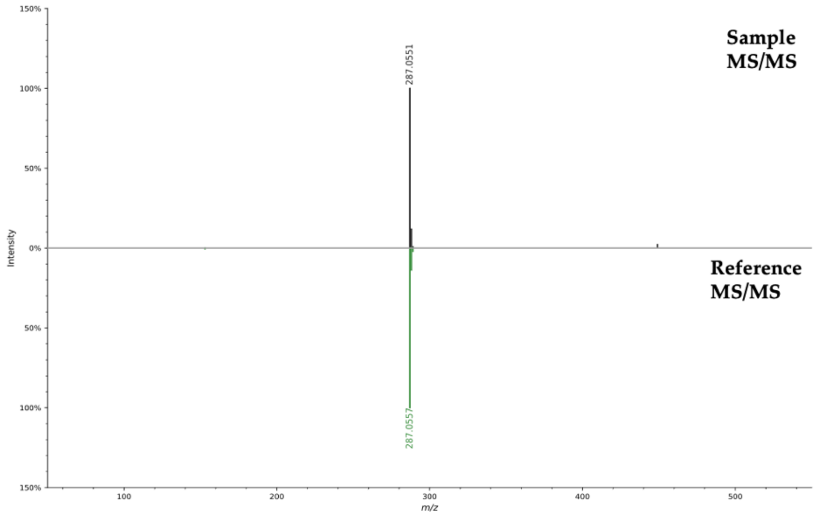

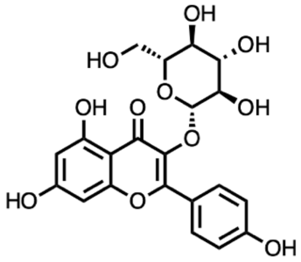

Spectral Match to Kaempferol-3-O-Glucoside
Cosine: 0.97, ppm error: 2.98

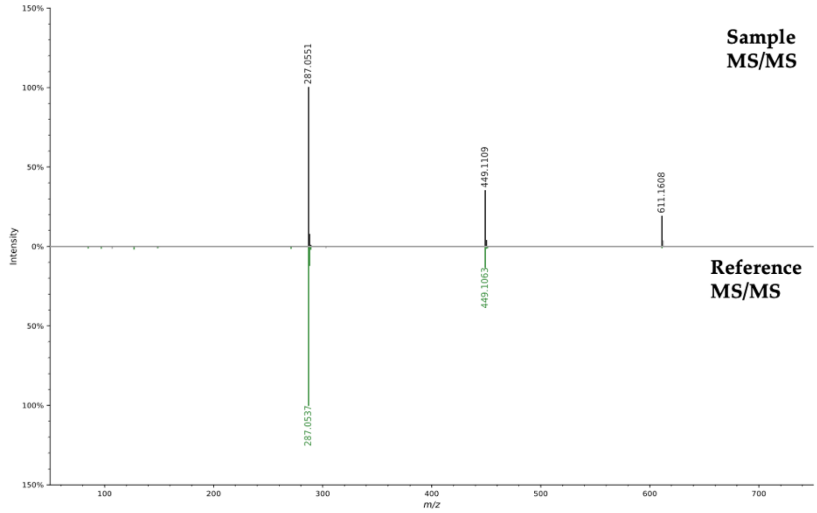

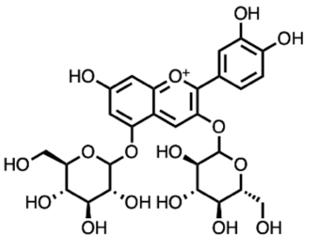

Spectral Match to Cyandin-3,5-O-Diglucoside
Cosine: 0.87, ppm error: 0.23

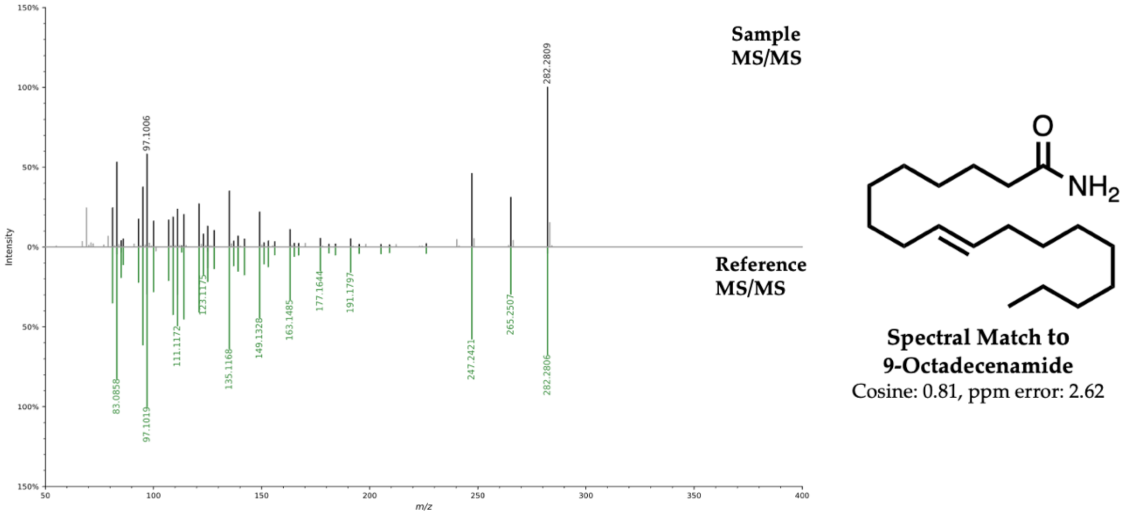

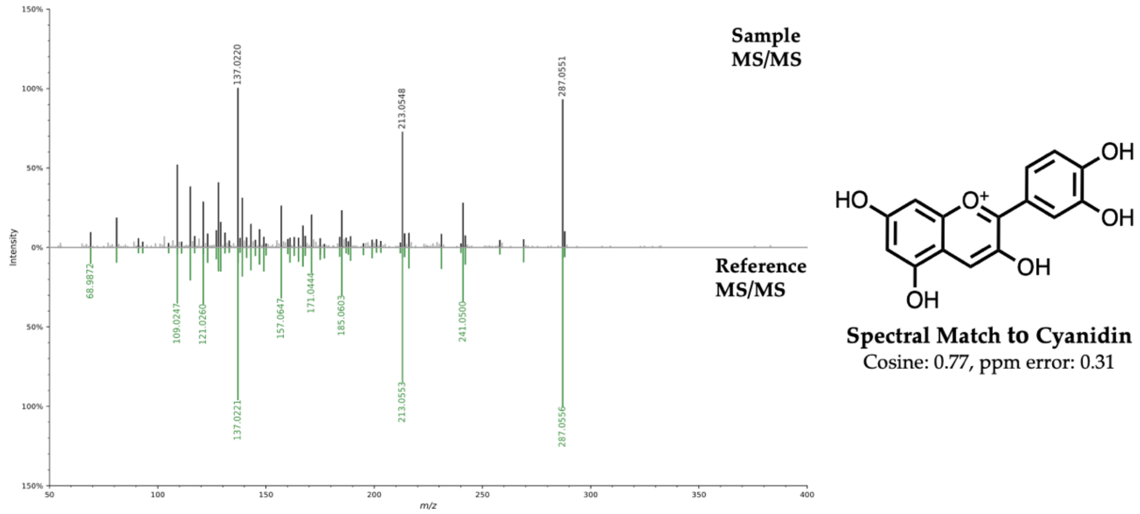

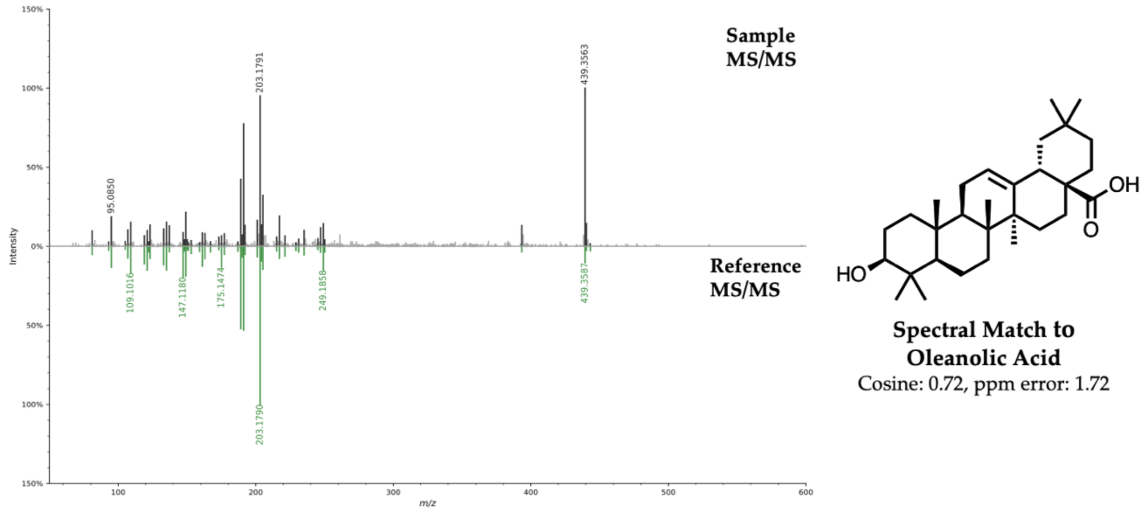

Figure S4. Tail-to-tail alignment of putatively identified metabolites from aqueous extract of *S. malaccense* fruit peel.

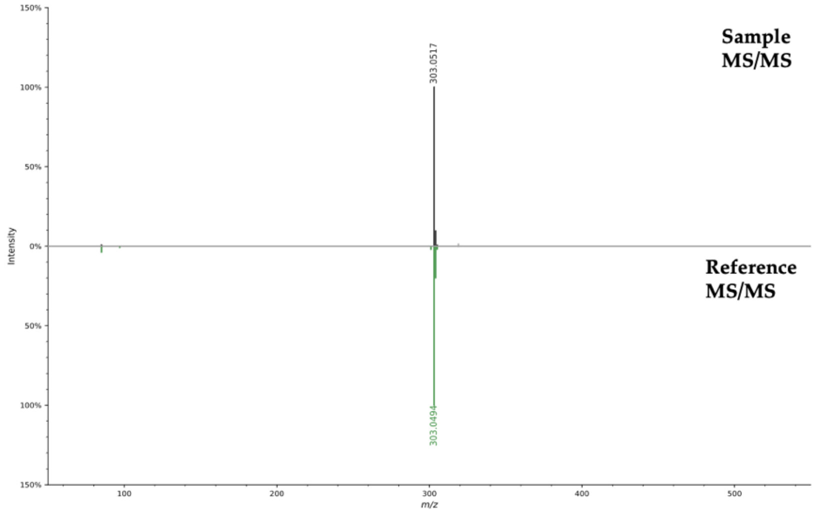

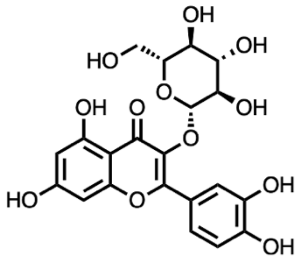

Spectral Match to Isoquercetin

Cosine: 0.96, ppm error: 1.18

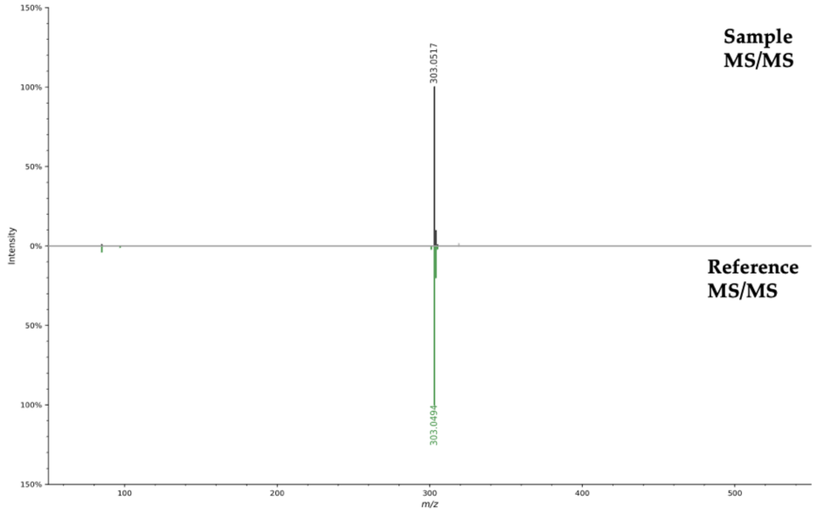

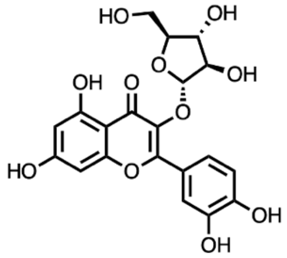

Spectral Match to Avicularin

Cosine: 0.94, ppm error: 2.96

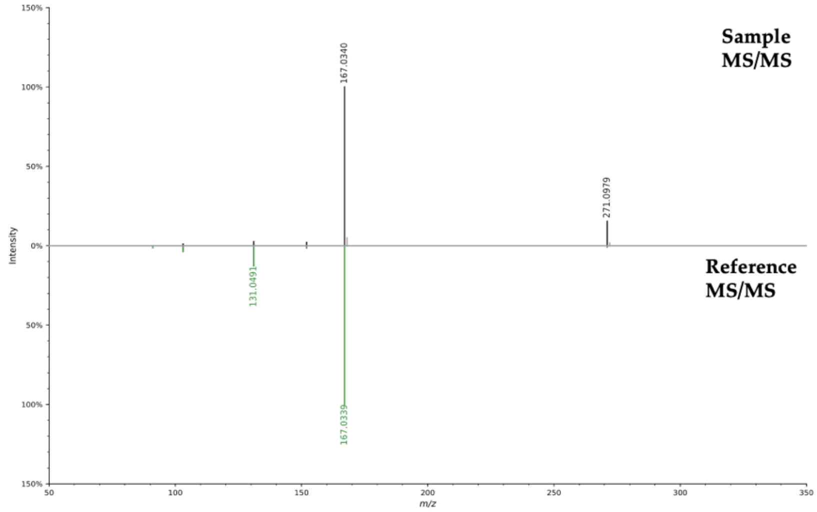

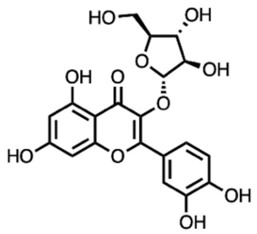

Spectral Match to Alpinetin
Cosine: 0.92, ppm error: 3.65

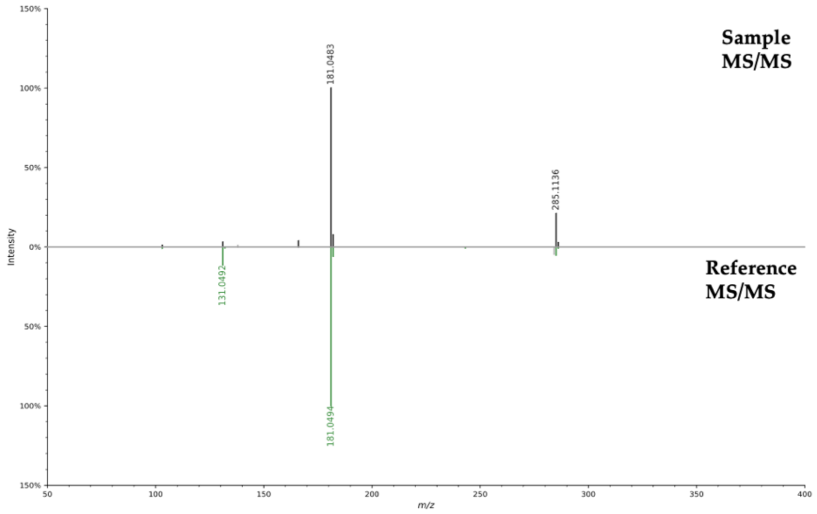

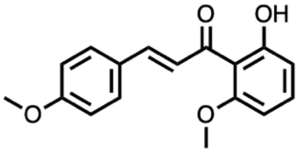

Spectral Match to 4',6'-Dimethoxy-2'-hydroxychalcone
Cosine: 0.92, ppm error: 0.21

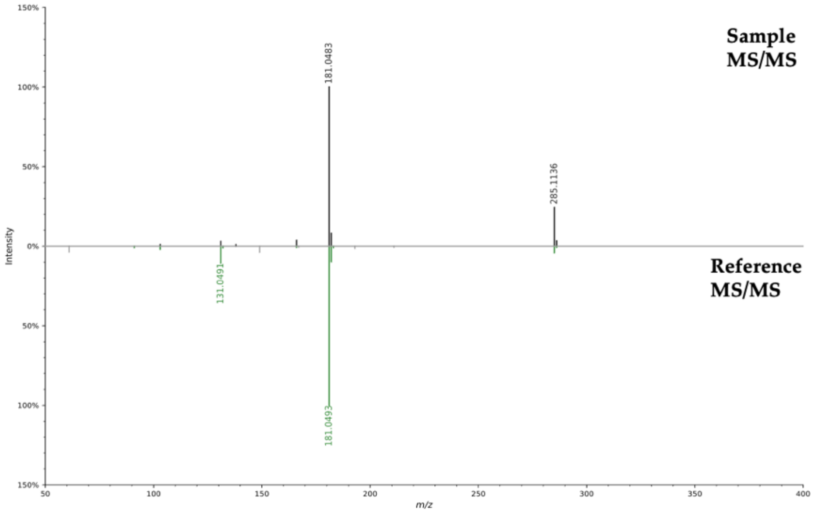

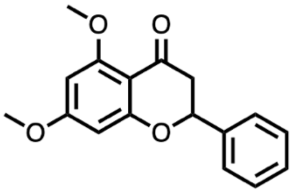

Spectral Match to Alpinetin Methyl Ether
Cosine: 0.92, ppm error: 0.21

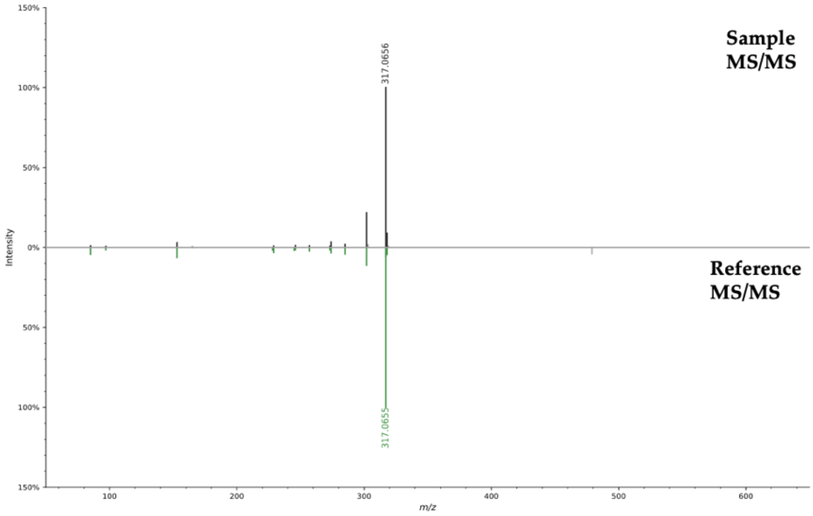

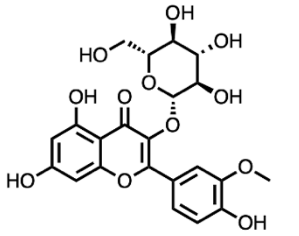

Spectral Match to Isorhamnetin-3-O-glucoside
Cosine: 0.90, ppm error: 3.55

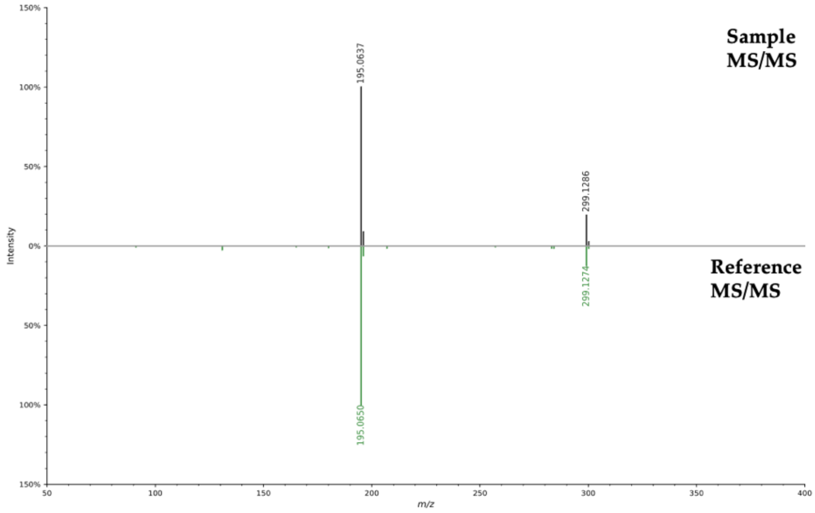

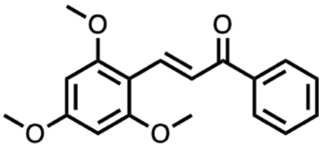

Spectral Match to 2,4,6-Trimethoxychalcone
Cosine: 0.90, ppm error: 2.71

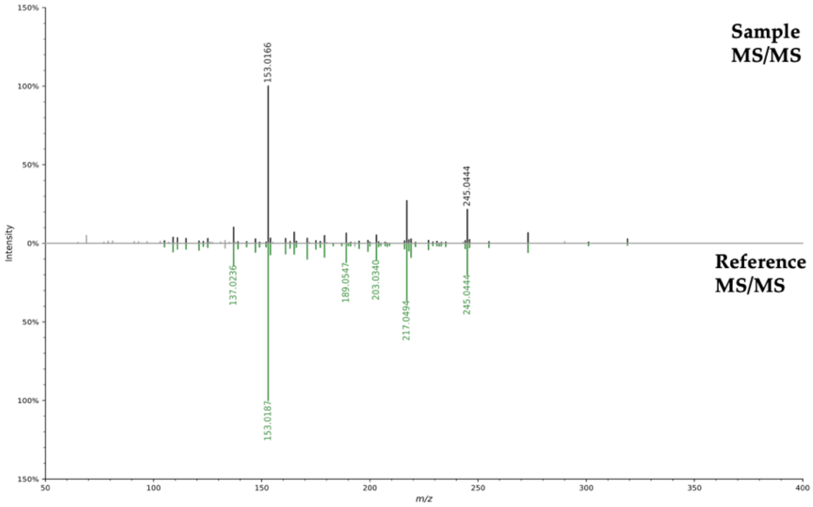

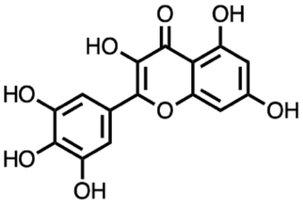

Spectral Match to Myricetin
Cosine: 0.87, ppm error: 4.83

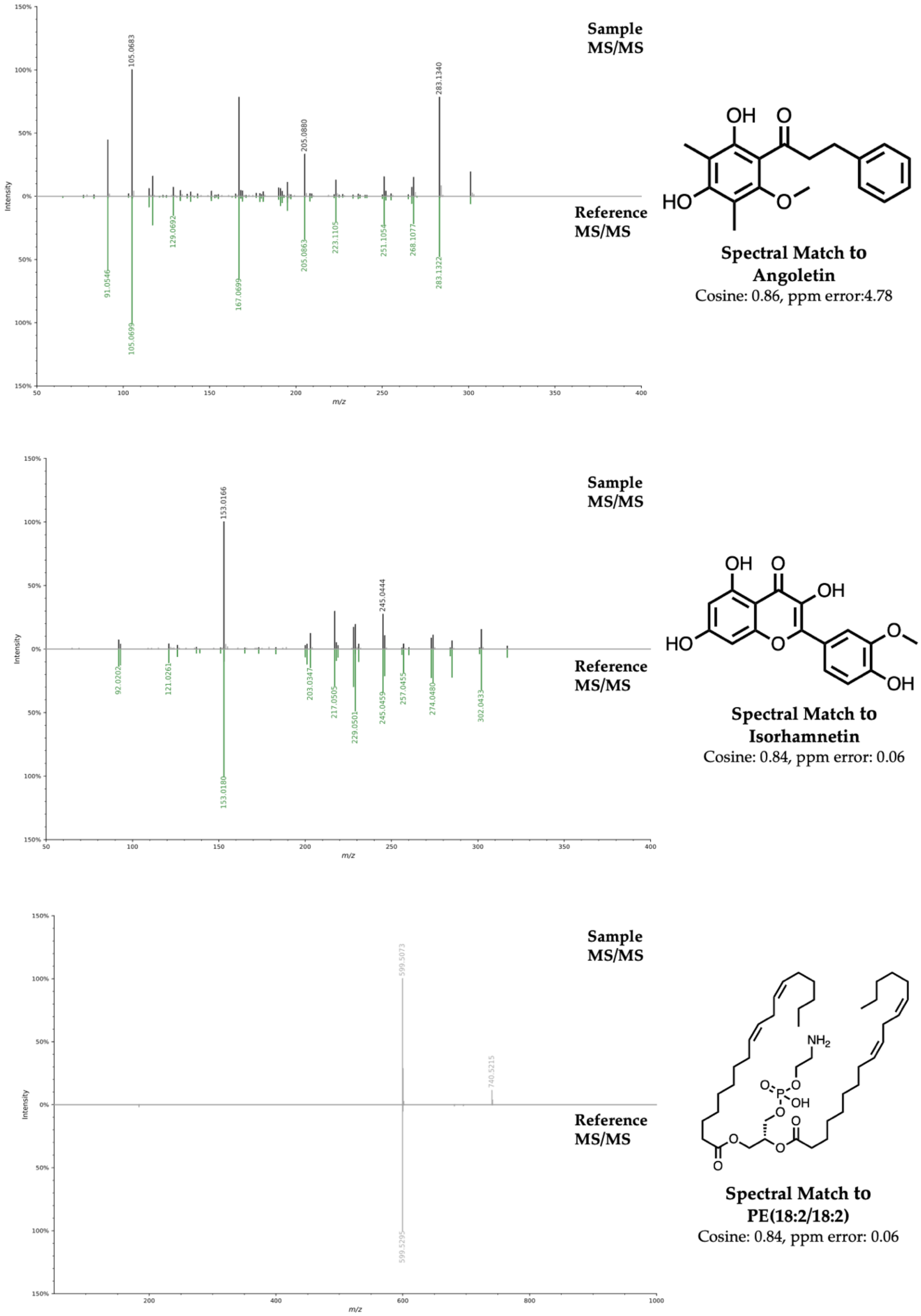

Figure S5. Tail-to-tail alignment of putatively identified metabolites from aqueous extract of *S. samarangense* fruit peel.

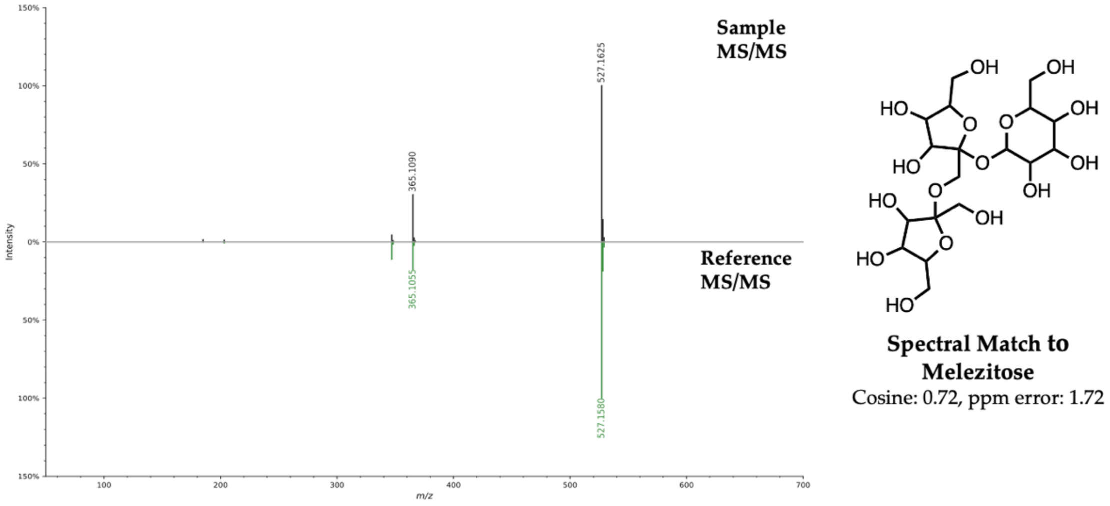

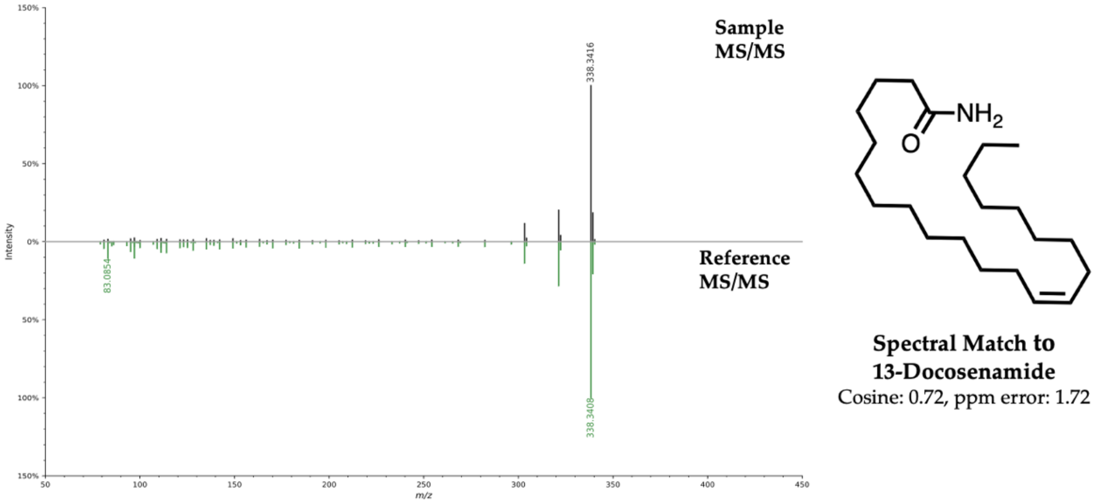

Figure S6. Tail-to-tail alignment of putatively identified metabolites from aqueous extracts of *E. deglupta* bark *and D. discolor* fruit peel.

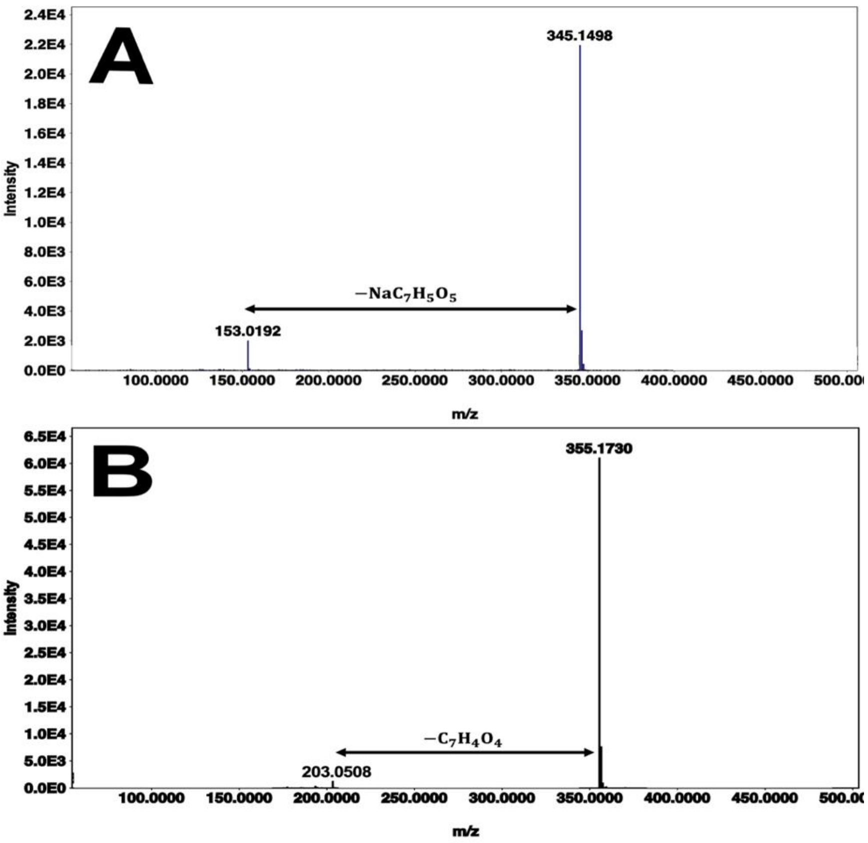

Figure S7. Tandem MS/MS spectra for digallic acid and glucogallin. Fragmentation pattern of sodiated precursor ions were obtained at 30-45 eV collision energy. Digallic acid (A) and glucogallin (B) gave rise to the gray and brown coloration of the extracted mabolo dye in the presence of different ionic mordants.

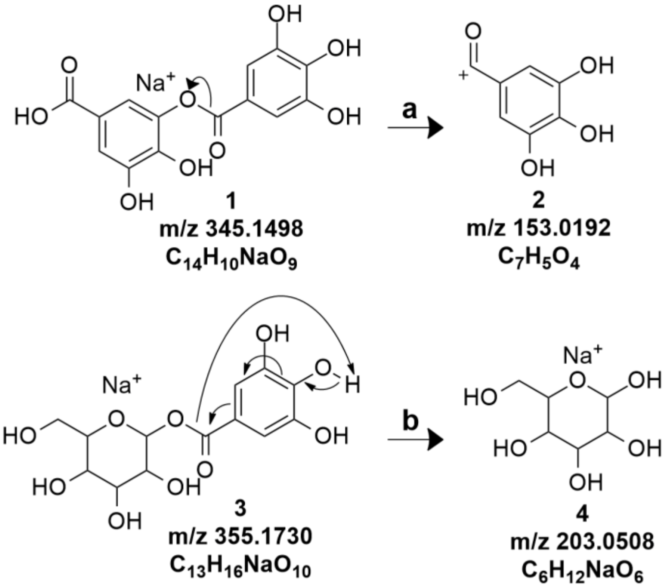

Figure S8. Proposed fragmentation scheme for sodiated ions of digallic acid and glucogallin. For the reactions, (a) loss of $NaC_7H_5O_5$ via inductive cleavage and (b) loss of galloyl substituent via remote H-rearrangement.

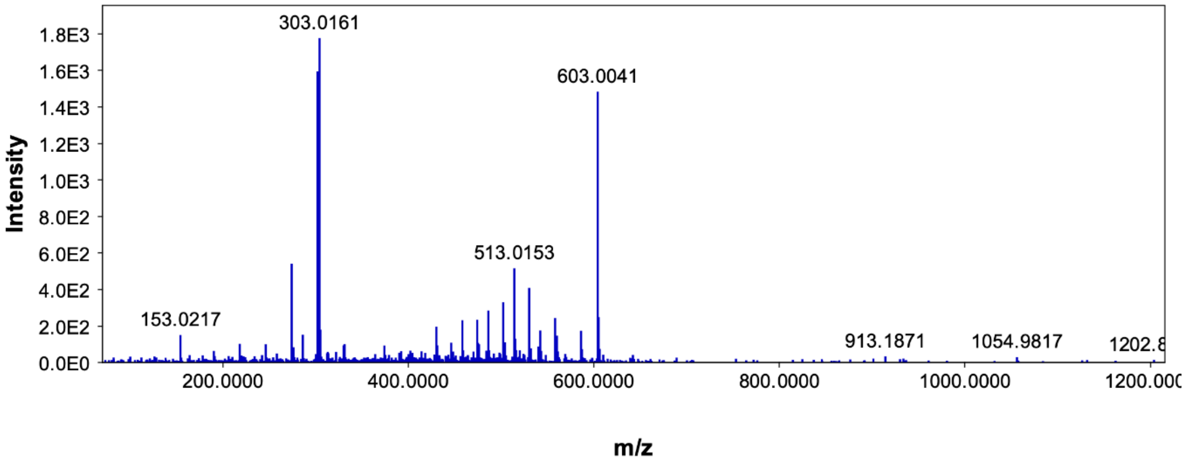

Figure S9. Tandem-MS spectrum for *m/z* 603.0041 associated with terminalin from *Terminalia catappa* leaf.

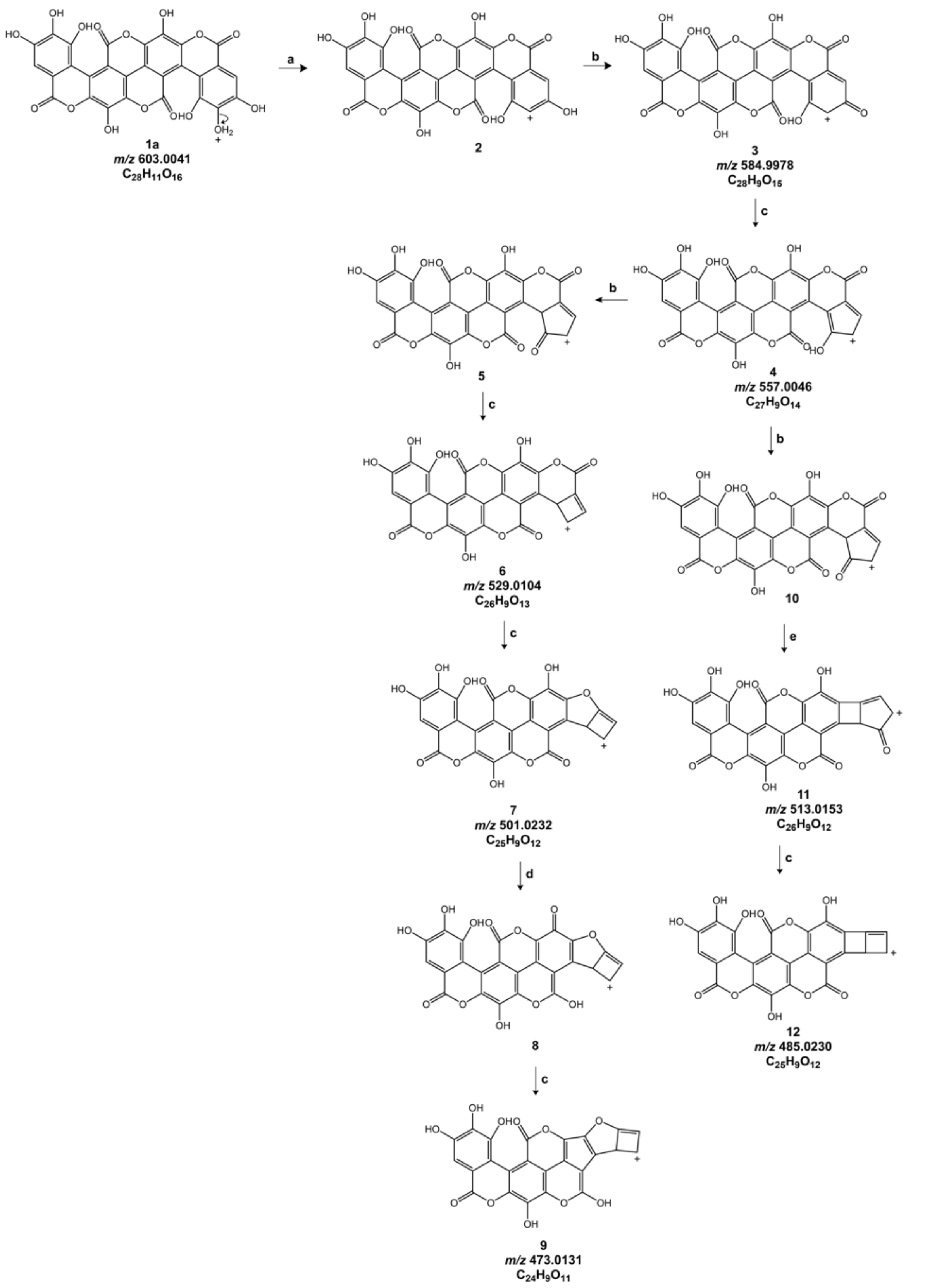

Figure S10. Proposed fragmentation scheme for *m/z* 603.0041 annotated as terminalin. For the reactions, (a) H^+ transfer, (b) Loss of H_2O through inductive cleavage, (c) loss of CO, (d) 1,7-sigmatropic H-shift, (e) loss of CO_2.

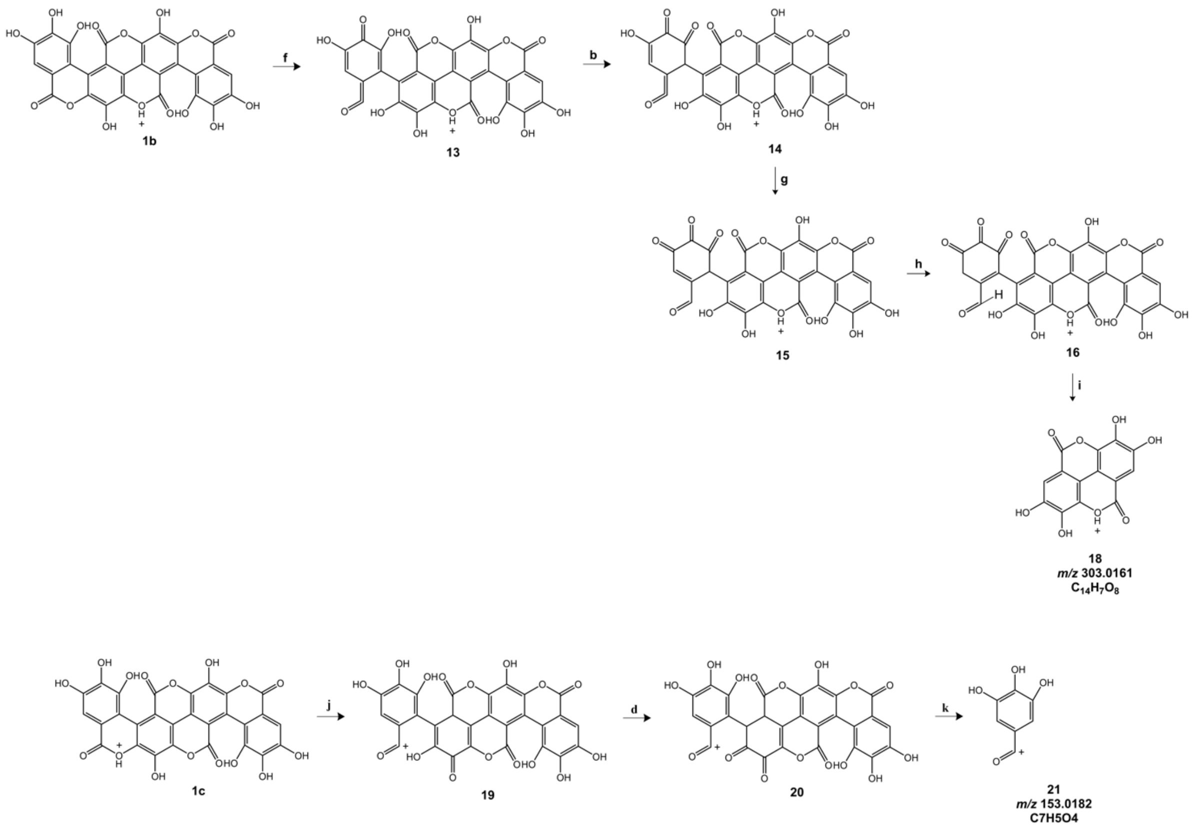

Figure S10 (cont.). Proposed fragmentation scheme for *m*/*z* 603.0041 annotated as terminalin. For the reactions, (a) H^+ transfer, (b) Loss of H_2O through inductive cleavage, (c) loss of CO, (d) 1,7-sigmatropic H-shift, (e) loss of CO_2, (f) preliminary formation of galloyl group, (g) 1,3-sigmatropic H-shift, (h) 1,5-sigmatropic H-shift, (i) loss of galloyl group, (j) inductive cleavage, (k) remote H-rearrangement.

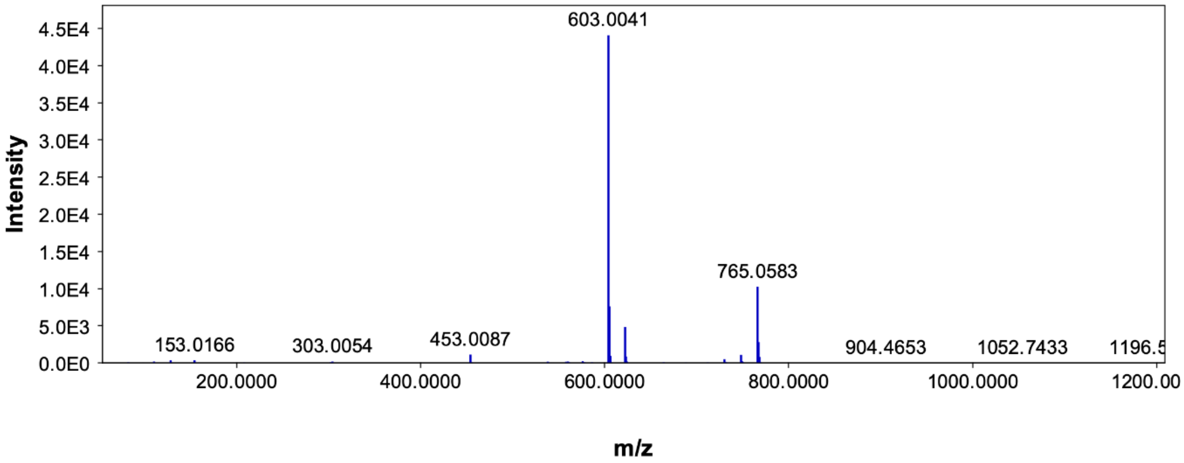

Figure S11. Tandem-MS spectrum for *m*/*z* 765.0583 associated with terminalin-O-glucoside from *Terminalia catappa* leaf.

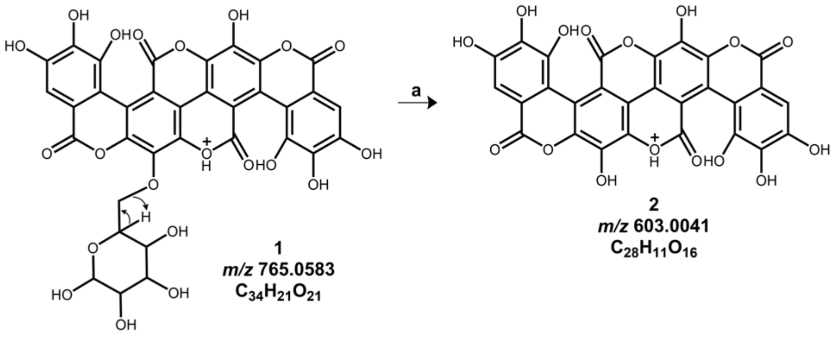

Figure S12. Proposed fragmentation scheme for *m/z* 765.0583 annotated as terminalin-O-glucoside. Reaction (a) correspond to remote H-rearrangement reaction resulting in the elimination of glucopyranoside.

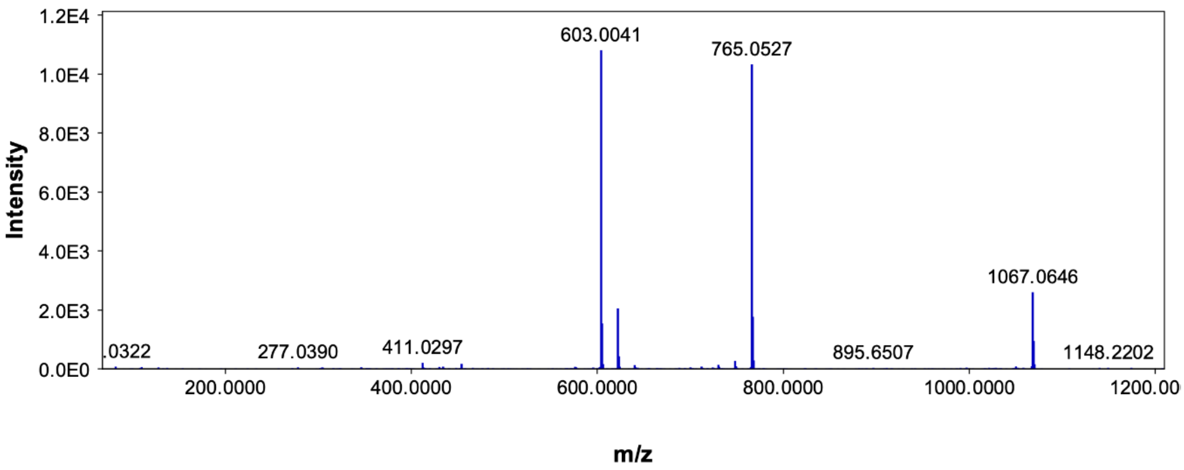

Figure S13. Tandem-MS spectrum for *m/z* 1067.0646 associated with terminalin-O-glucoside-O-hexahydroxydiphenic acid from *Terminalia catappa* leaf.

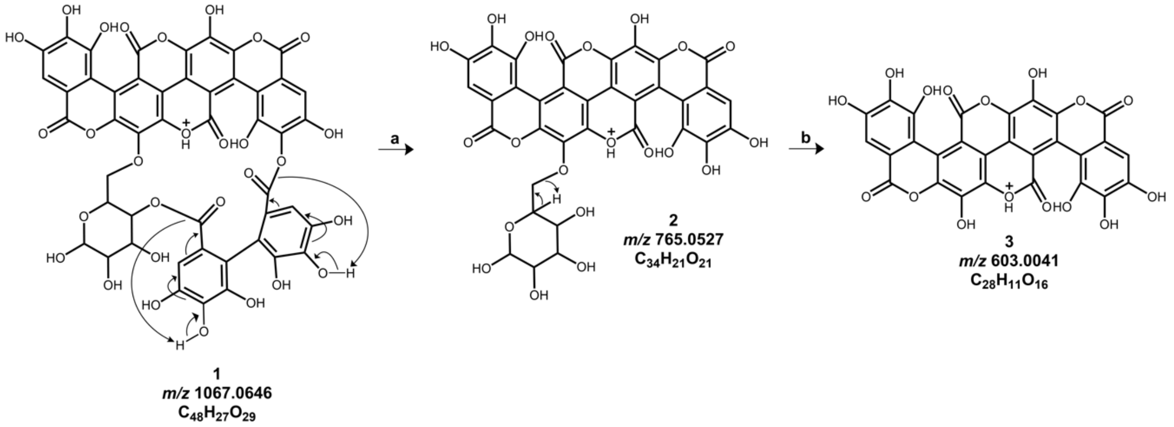

Figure S14. Proposed fragmentation scheme for *m/z* 765.0583 annotated as terminalin-O-glucoside O-hexahydroxydiphenic acid. Reaction (a) correspond to loss of hexahydroxydiphenic acid (-302 Da) while (b) is remote H-rearrangement reaction resulting in the elimination of glucopyranoside.

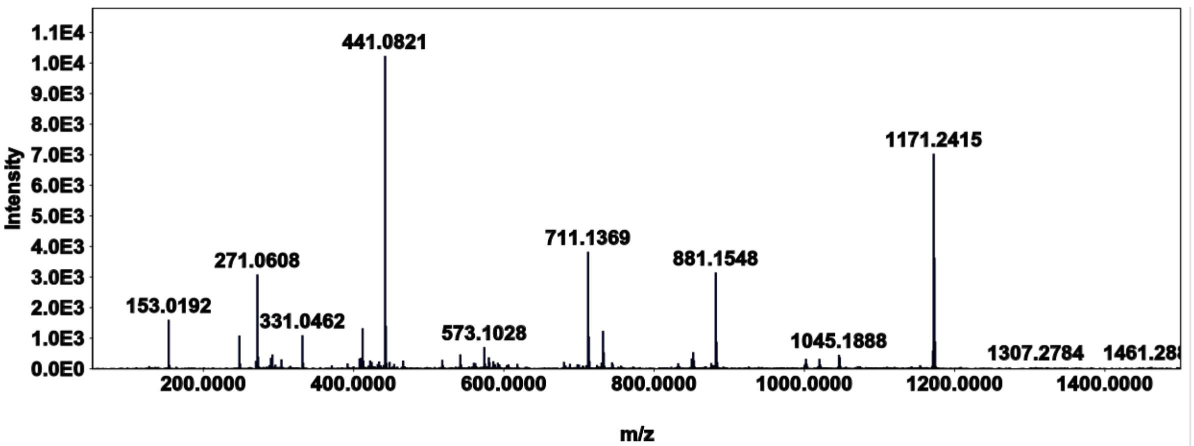

Figure S15. Tandem MS/MS spectra of *m/z* 1171.2415 annotated as an epicatechin gallate trimer. Collision induced dissociation (CID) at 30-45 eV collision energy in the positive ionization mode produced product ions that lead to the characterization of the proanthocyanidin.

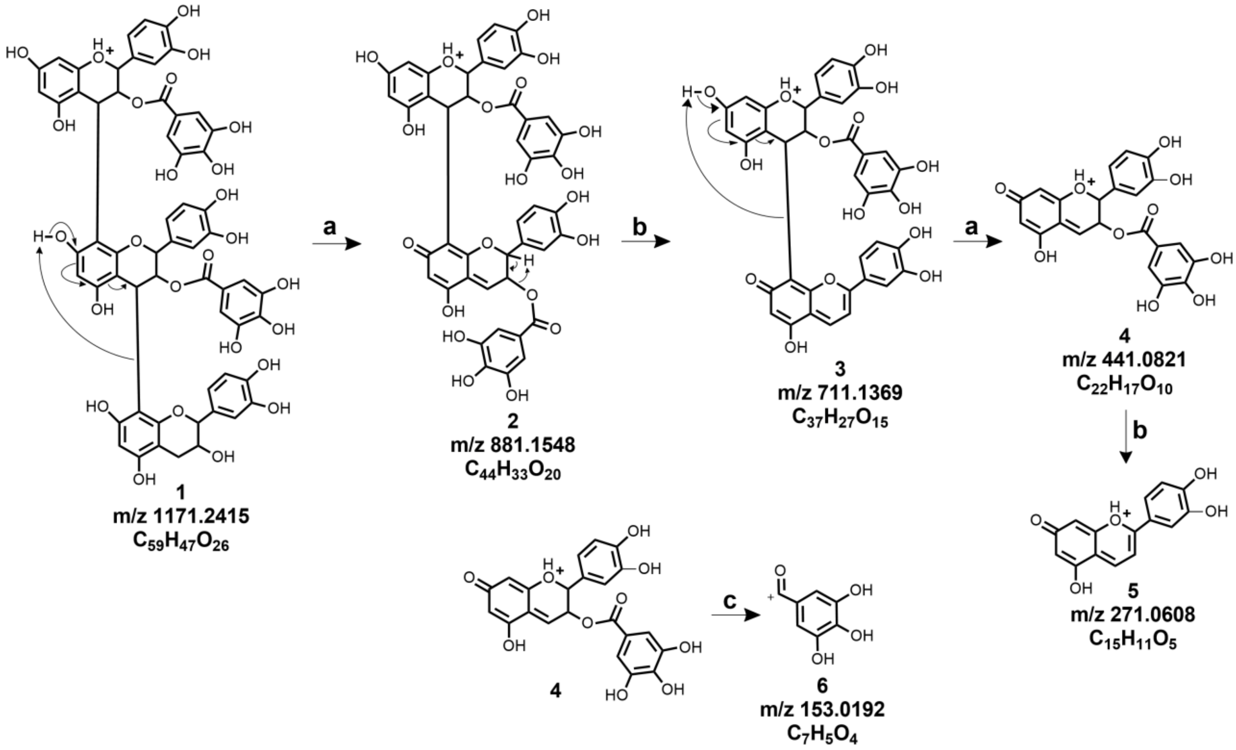

Figure S16. Theoretical fragmentation of (Epi)catechin gallate - (Epi) catechin gallate – (Epi)catechin gallate. Theoretical mass of expected product ions is comparable to the experimental mass and supports the annotation of m/z 1171.2415 as the mentioned proanthocyanidin. For the reactions (a) quinone-methide elimination (b) loss of gallic acid via remote H-rearrangement (c) inductive cleavage.

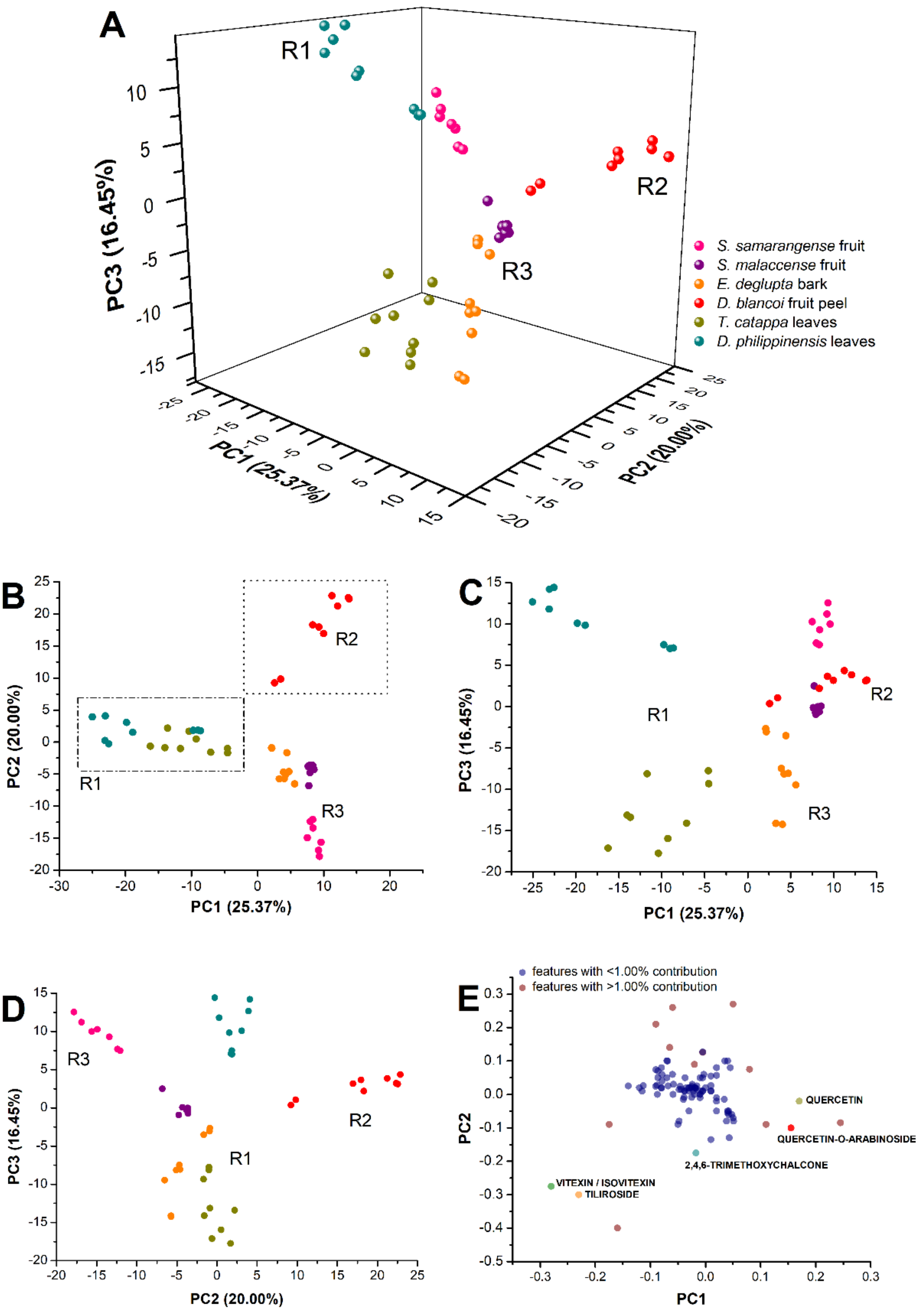

Figure S17. PCA scores plots of all dye extracts. Three(3)-dimensional scores (A) show spread of data in three regions (R1, R2, and R3) as shown in Figure 9 of the article. Different perspectives are shown in B (PC1 vs PC2), C (PC1 vs PC3) and D (PC2 and PC3). Regions are labelled accordingly. PC1 vs PC2 loadings plot shown in E highlights features contributing primarily to the variance.

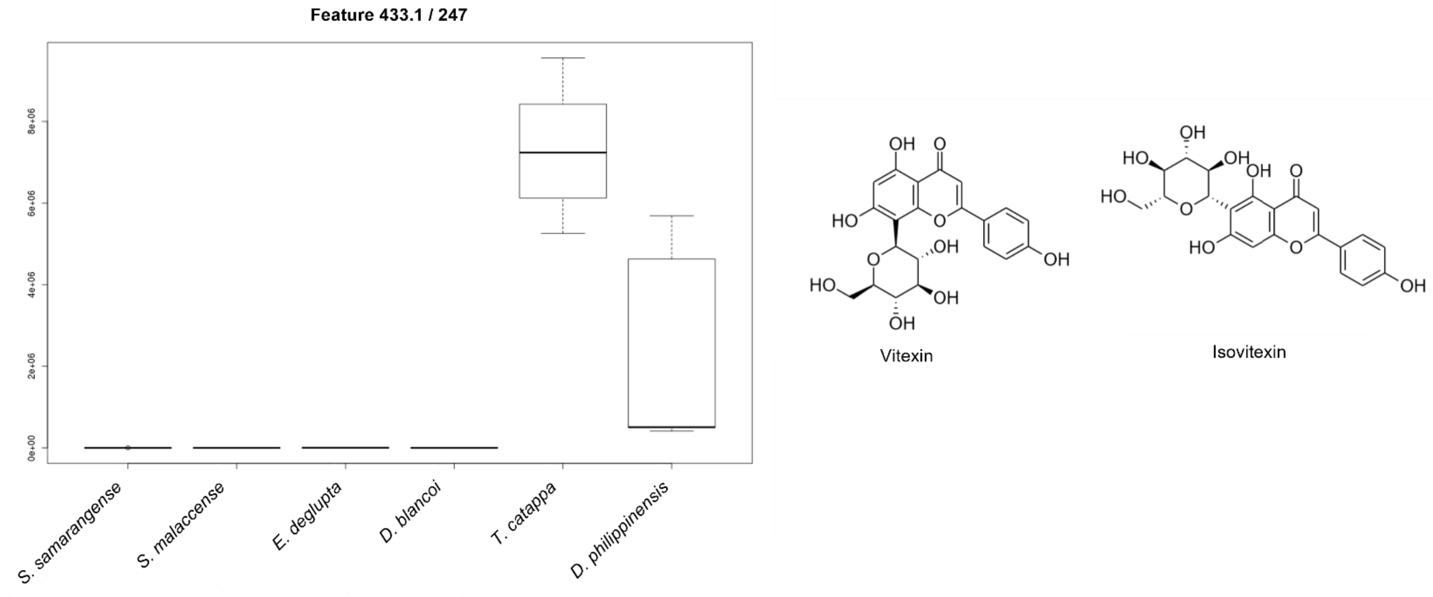

Figure S18. Box-and-whisker plot corresponding to vitexin/isovitexin. This chemical feature is present only in *D. philippinensis* fruit peel and in the traditional dye source*, T. catappa.*

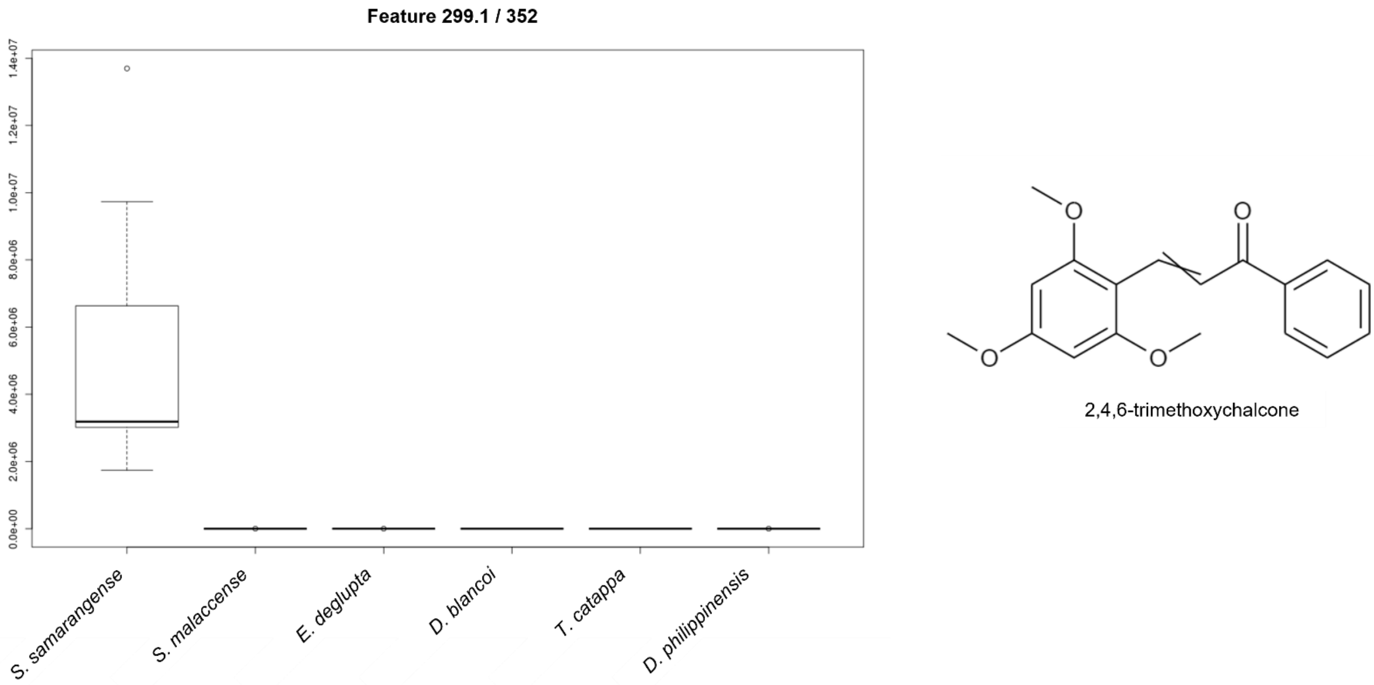

Figure S19. Box-and-whisker plot corresponding to 2,4,6-trimethoxychalcone.

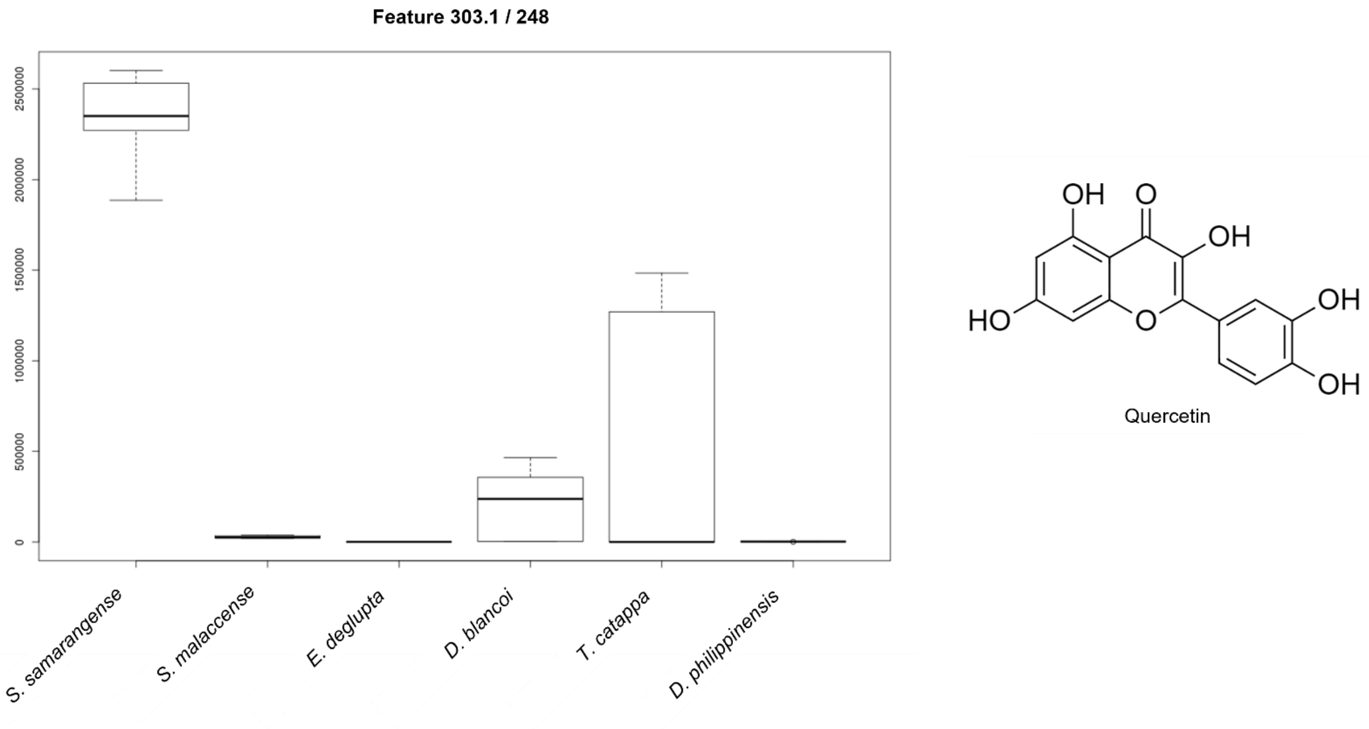

Figure S20. Box-and-whisker plot corresponding to quercetin. This chemical feature is highly abundant in *S. samarangense* fruit peel.

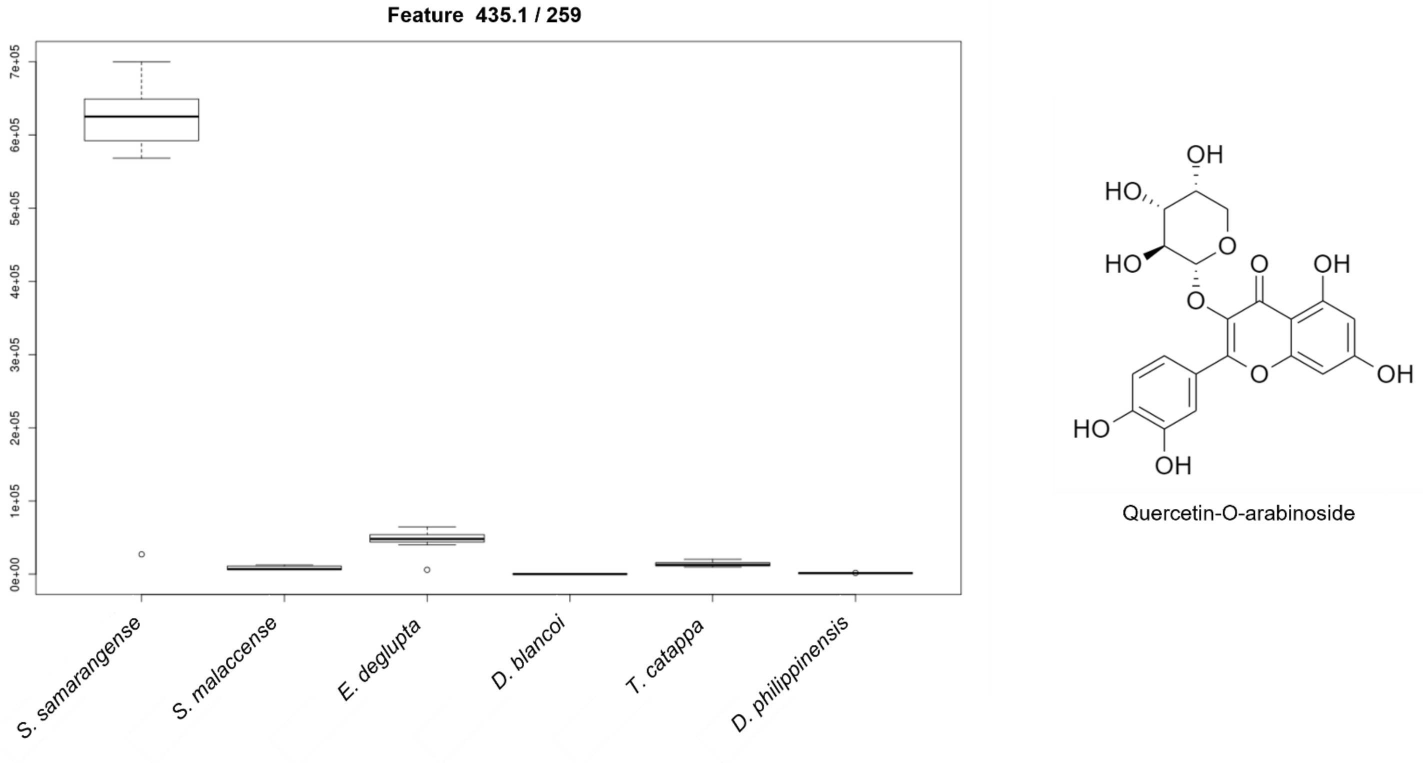

Figure S21. Box-and-whisker plot corresponding to quercetin-O-arabinoside. This chemical feature is highly abundant in *S. samarangense* fruit peel.

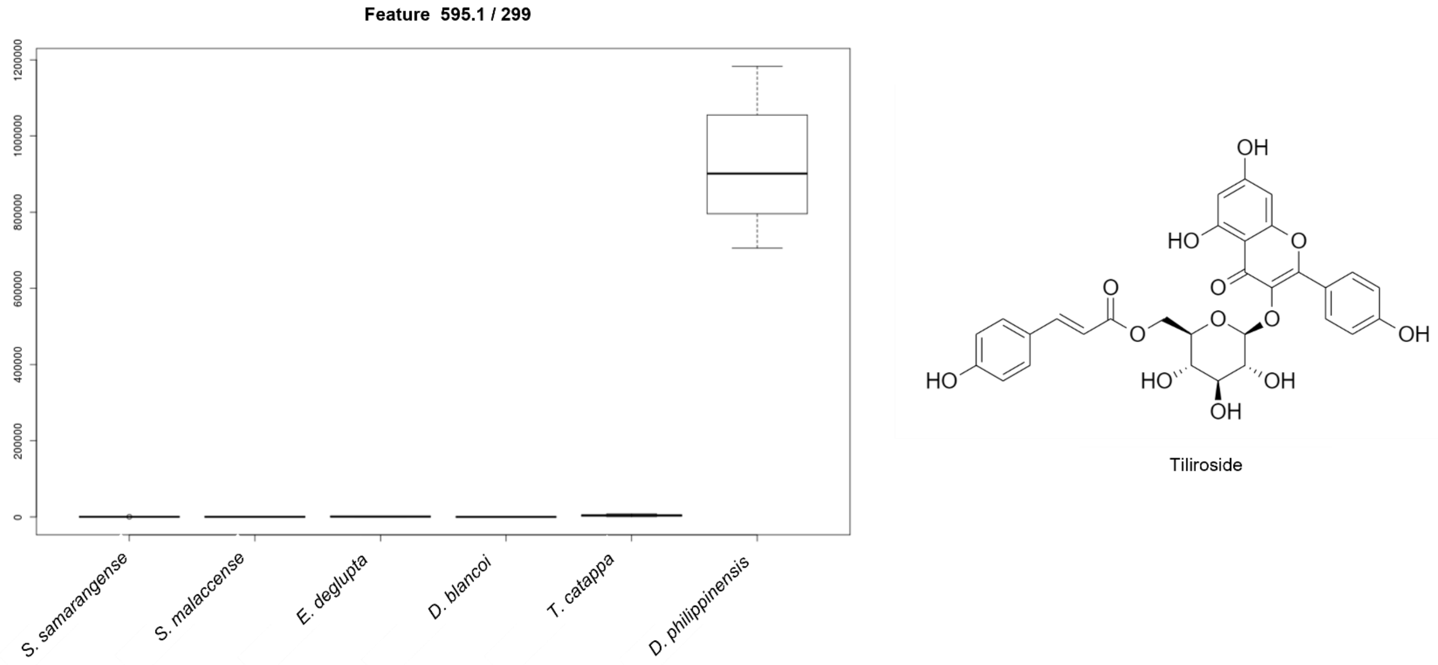

Figure S22. Box-and-whisker plot corresponding to tiliroside. This chemical feature is highly abundant in *D. philippinensis* fruit peel.

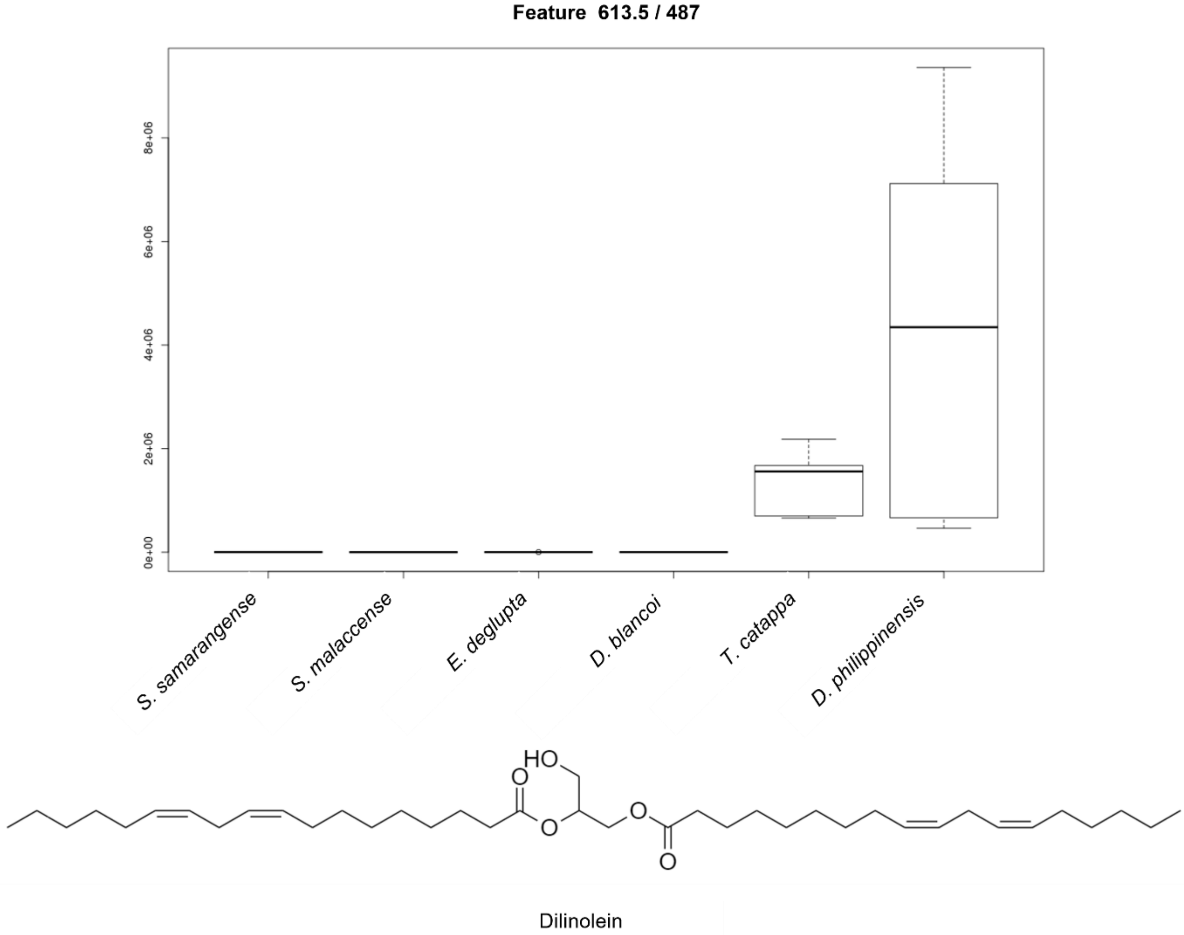

Figure S23. Box-and-whisker plot corresponding to dilinolein.

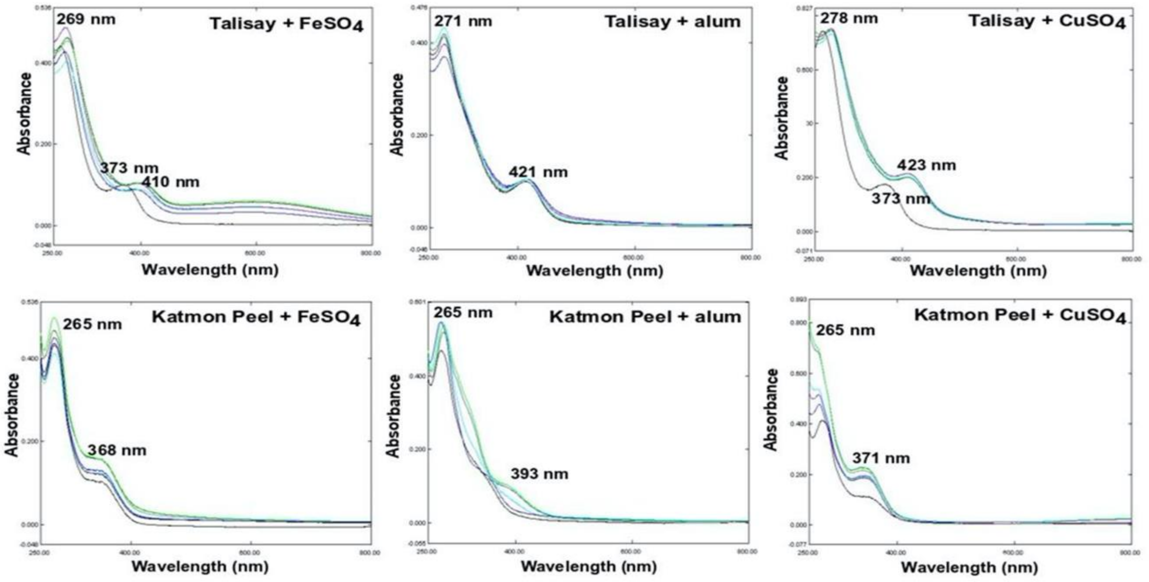

Figure S24. Comparison of the UV-Vis spectra of *T. catappa* leaf and *D. philippinensis* fruit peel in the presence of $FeSO_4$, alum, and $CuSO_4$ mordant. Absorption of the band II of flavonoid were observed at 368-373 nm and was very prominent at non-mordanted extract. Bathochromic and hyperchromic shift were noted in the presence of mordants.

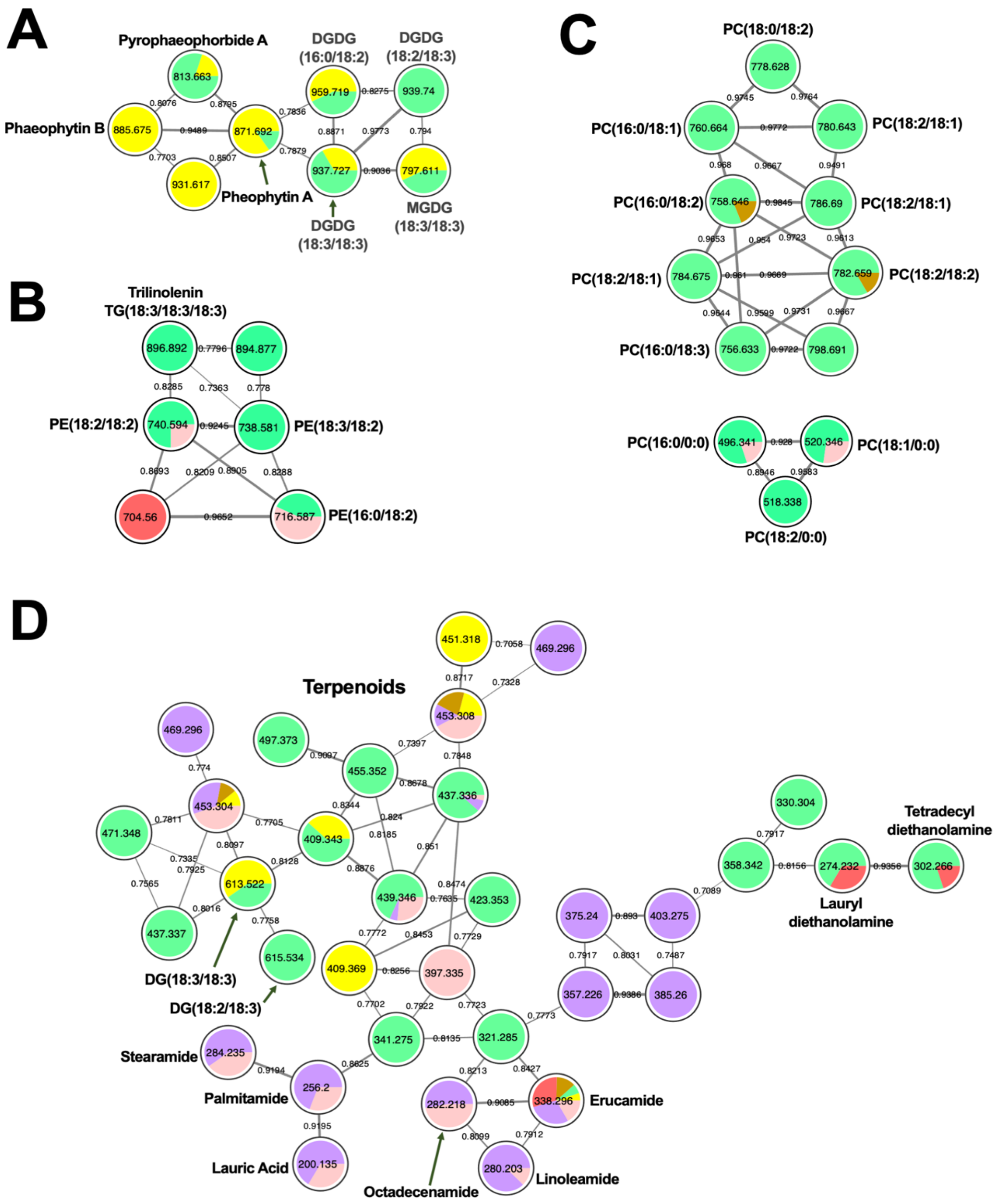

Figure S25. Molecular networks of non-pigment metabolites from the extracted samples. Chlorophyll pigments and catabolites, together with sodiated glycerolipids clustered in (a). Phosphoethanolamine and phosphocholine type lipids were identified on (b) and (c), respectively. On the other hand, fatty acids and terpenoids clustered on (d).
